# Binding of galectin-1 to integrin β1 potentiates drug resistance by promoting survivin expression in breast cancer cells

**DOI:** 10.18632/oncotarget.16208

**Published:** 2017-03-15

**Authors:** KeeSoo Nam, Seog-ho Son, Sunhwa Oh, Donghwan Jeon, Hyungjoo Kim, Dong-Young Noh, Sangmin Kim, Incheol Shin

**Affiliations:** ^1^ Department of Life Science, Hanyang University, Seoul, 133-791, Korea; ^2^ Cancer Research Institute, Seoul National University College of Medicine, Seoul, 110-744, Korea; ^3^ Department of Surgery, Samsung Medical Center, Seoul, 135-710, Korea; ^4^ Natural Science Institute, Hanyang University, Seoul, 133-791, Korea

**Keywords:** galectin-1, integrin β1, STAT3, survivin, drug resistance

## Abstract

Galectin-1 is a β-galactoside binding protein secreted by many types of aggressive cancer cells. Although many studies have focused on the role of galectin-1 in cancer progression, relatively little attention has been paid to galectin-1 as an extracellular therapeutic target. To elucidate the molecular mechanisms underlying galectin-1-mediated cancer progression, we established galectin-1 knock-down cells via retroviral delivery of short hairpin RNA (shRNA) against galectin-1 in two triple-negative breast cancer (TNBC) cell lines, MDA-MB-231 and Hs578T. Ablation of galectin-1 expression decreased cell proliferation, migration, invasion, and doxorubicin resistance. We found that these effects were caused by decreased galectin-1-integrin β1 interactions and suppression of the downstream focal adhesion kinase (FAK)/c-Src pathway. We also found that silencing of galectin-1 inhibited extracellular signal-regulated kinase (ERK)/signal transducer and activator of transcription 3 (STAT3) signaling, thereby down-regulating survivin expression. This finding implicates STAT3 as a transcription factor for survivin. Finally, rescue of endogenous galectin-1 knock-down and recombinant galectin-1 treatment both recovered signaling through the FAK/c-Src/ERK/STAT3/survivin pathway. Taken together, these results suggest that extracellular galectin-1 contributes to cancer progression and doxorubicin resistance in TNBC cells. These effects appear to be mediated by galectin-1-induced up-regulation of the integrin β1/FAK/c-Src/ERK/STAT3/survivin pathway. Our results imply that extracellular galectin-1 has potential as a therapeutic target for triple-negative breast cancer.

## INTRODUCTION

Triple-negative breast cancers (TNBCs) are defined as a subset of breast tumors with absent or low levels of estrogen receptor (ER), progesterone receptor (PR), and HER2 expression [1]. Since they lack expression of these three receptors, patients with TNBC have limited treatment options and short life expectancies and are associated with poor prognosis [2, 3]. Therefore, there is an urgent need to identify new target molecules for more effective and sustainable therapies for treating TNBC.

Galectin-1, encoded by the *LGALS1* gene, was first identified as a β-galactoside binding protein [4]. Galectin-1 has a conserved carbohydrate-recognition domain (CRD) consisting of about 130 amino acids that mediates binding to carbohydrate-rich regions of cell surface proteins [4, 5]. Moreover, galectin-1 is involved in cell transformation via direct interactions with cell surface oncogenic proteins such as integrins, laminin, and fibronectin, leading to subsequent cancer progression [6–8]. In addition, many studies have investigated the function of galectin-1 in the immunosuppressive mechanisms of human melanoma [9], neuroblastoma [10], and pancreatic carcinoma [11]. However, few reports have investigated the potential of galectin-1 as an extracellular therapeutic target, primarily because galectin-1 is predominantly a secretory protein. Therefore, we were motivated to investigate the potential of galectin-1 as a TNBC-specific extracellular therapeutic target molecule, even though galectin-1 is a typical secretory protein.

Integrins are typical cell adhesion receptors related to cell proliferation, migration, invasion, and adhesion in various cancer cells [12–15]. The integrin family consists of 24 αβ heterodimeric groups. The α subunit determines the binding specificity of a given integrin to its cognate ligands, whereas the β subunit drives numerous downstream signaling through interactions with the cytoskeleton [16]. Interestingly, the integrin β1 subunit has been reported to bind galectin-1 directly and to activate cytoskeletal-associated focal adhesion kinase (FAK) [7]. Activation of FAK, in turn, induces downstream c-Src or ERK signaling-mediated cell proliferation, migration, invasion, and adhesion in various cancer cells [17–20].

The transcription factor signal transducer and activator of transcription 3 (STAT3) is well known to play crucial roles in immunosuppression and tumorigenesis [21–25]. STAT3 is activated by diverse growth factors, hormones, and cytokines. After phosphorylation of Tyr705, STAT3 forms a dimer and translocates to the nucleus, where it acts as a transcription factor [26]. Tyr705 phosphorylation of STAT3 is mediated by tyrosine kinases such as EGFR [27], JAK [28], and c-Src [29] and activation of downstream signaling results in cell proliferation, migration, and invasion [30]. Nuclear STAT3 binds to the consensus sequences of promoter regions of target genes such as c-Fos, HIF-1α, c-Myc, Twist, and survivin, thereby driving their transcription [30–35]. STAT3 can also be phosphorylated at Ser727 by extracellular signal-regulated kinase (ERK), which augments the effect of Tyr705 phosphorylation [36]. However, the precise role of Ser727-phosphorylated STAT3 remains controversial.

Survivin is a 16.5 kDa protein that is classified as a member of the inhibitor of apoptosis protein (IAP) family of anti-apoptotic proteins [37]. Survivin can bind caspase-3, a protease effector of cell death, thereby inhibiting its activity [38]. Survivin has been shown to be abundantly expressed in many human cancers [37], and its expression is increased by many transcription factors, including Sp1 [39], HIF-1α [40], Egr-1 [41], and STAT3 [35].

We found that galectin-1 drives doxorubicin resistance via direct interaction with integrin β1, which in turn activates FAK/c-Src/ERK/STAT3 signaling. This phenomenon culminates in nuclear translocation of STAT3, a transcription factor driving survivin expression, in triple-negative breast cancer cells.

## RESULTS

### Galectin-1 is overexpressed in patients with triple negative breast cancer and ablation of galectin-1 decreases secretion and cell surface level of galectin-1

**Figure 1:**
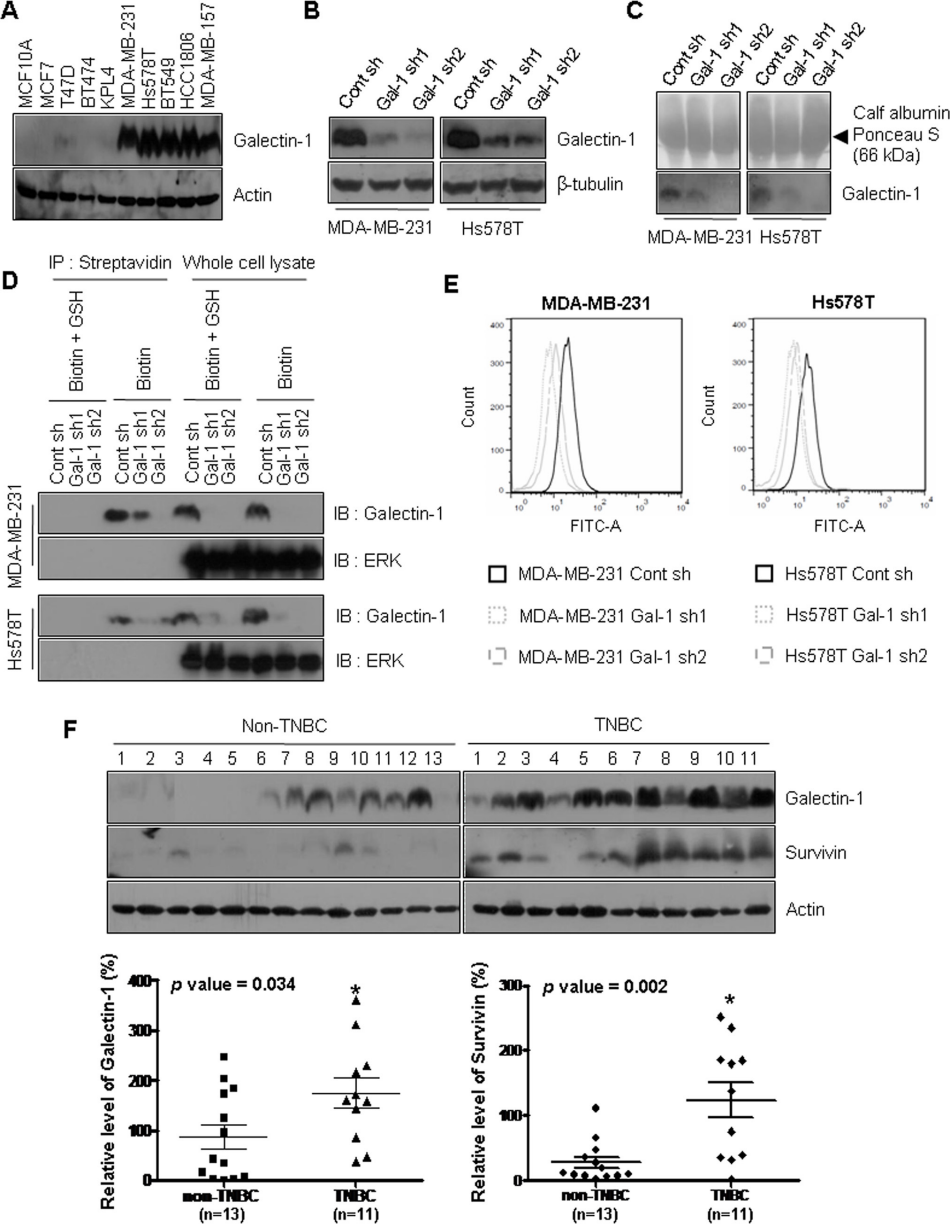
Galectin-1 is overexpressed in specimens from patients with triple negative breast cancer and ablation of galectin-1 decreases secretion and cell surface level of galectin-1 (**A**) Galectin-1 protein expression was analyzed in the human breast cancer cell lines MDA-MB-231, Hs578T, BT474, T47D, and MCF7 by western blotting. (**B**) The efficiency of galectin-1 silencing with different human galectin-1-targeting sequences was confirmed by western blot analysis (MDA-MB-231, Gal-1 sh1 and Gal-1 sh2; Hs578T, Gal-1 sh1 and Gal-1 sh2). Scrambled shRNA was also used as a control (MDA-MB-231 Cont sh and Hs578T Cont sh). (**C**) Ablation of galectin-1 secretion into culture medium was detected by TCA/acetone precipitation of proteins. Equal protein loading of each lane was confirmed by Ponceau staining. (**D**) Silencing of cell surface galectin-1 was confirmed by biotinylation assays. Cells were incubated with 0.5 mg/ml EZ-Link NHS-SS-Biotin for 30 min at 4°C. The biotinylated proteins were captured with streptavidin and analyzed by western blotting using anti-galectin-1 antibody. ERK was used as an endogenous negative control. (**E**) Galectin-1 knock-down was confirmed by flow cytometry cell surface labeling experiments with anti-galectin-1 antibody. Experiments were performed in triplicate, and a representative histogram is shown. (**F**) Western blot analysis showing the expression levels of galectin-1 and survivin in lysates of primary tumor specimens from patients with breast cancer (*n* = 24). Galectin-1 and survivin expression were significantly up-regulated in patients with TNBC compared to patients with non-TNBC. Error bars represent means ± SD of all experiments (*p < 0.05).

To investigate the effect of galectin-1 ablation on breast cancer cells, we established galectin-1 knock-down cells using two shRNA constructs recognizing different target sequences (Gal-1 sh1 and Gal-1 sh2). Cells expressing scrambled shRNA were used as controls (Cont sh). We observed higher levels of galectin-1 in the two triple-negative breast cancer (TNBC) cell lines, MDA-MB-231 and Hs578T, than in non-TNBC cell lines (Figure 1A). Based on this observation, we knocked down galectin-1 in MDA-MB-231 and Hs578T cells. We then confirmed that the amount of secreted galectin-1 was decreased in the culture medium of galectin-1 knock-down cells (Figure 1B, 1C). The cell surface protein biotinylation assay also showed that the level of cell surface galectin-1 was decreased in the galectin-1 knock-down cells. Cytosolic ERK was not detected by both cell surface labeling experiments (Figure 1D). In addition, flow cytometry analyses confirmed the decreased labeling of FITC in the cell surface of galectin-1 knock-down cells compared to control shRNA cells (Figure 1E). The results indicate that galectin-1 attached to other cell surface receptor protein after secretion.

Galectin-1 expression is elevated in highly metastatic human breast tumors [42] and has been shown to be correlated with poor prognosis in patients with aggressive breast cancer [43]. To confirm this relationship, we analyzed galectin-1 expression in breast cancer tissue specimens by western blotting (Supplementary Table 1). Galectin-1 was significantly up-regulated in patients with TNBC compared to patients with non-TNBC (Figure 1F). However, we could not observe galectin-1 expression in normal breast tissues obtained from non-TNBC or TNBC patients (Supplementary Figure 1).

### Ablation of galectin-1 decreases cell migration and invasion

**Figure 2:**
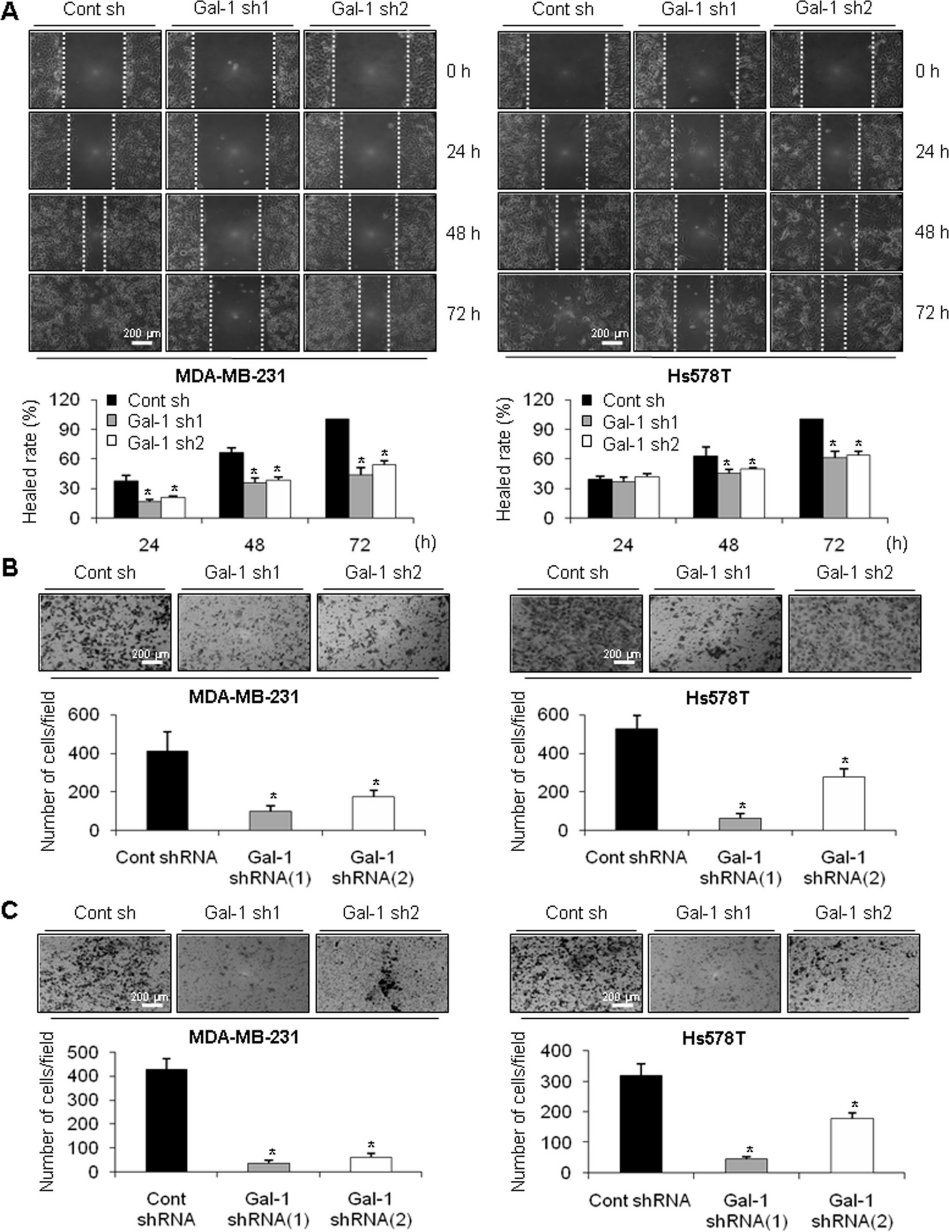
Effect of galectin-1 silencing on migration and invasion of human breast cancer cells (**A**) For the wound healing assay, cells were seeded at a density of 3 × 10^5^ cells/well in 6-well plates to achieve 95% confluence. A vertical or horizontal wound was created using a 10 ml pipette tip, after which the wounded cells were washed three times with PBS and FBS-free DMEM. Over 72 h, the wounded areas were photographed at the designated times to assess wound closure. Assays were carried out in triplicate. Error bars represent means ± SD of all experiments. *p* values (bar vs bar, left panel) : *p* = 0.003 (1 vs 2), *p* = 0.004 (1 vs 3), *p* = 0.002 (4 vs 5), *p* = 0.003 (4 vs 6), *p* = 0.0001 (7 vs 8), *p* = 0.002 (7 vs 9). *p* values (bar vs bar, right panel) : *p* = 0.04 (4 vs 5), *p* = 0.04 (4 vs 6), *p* = 0.0005 (7 vs 8), *p* = 0.0004 (7 vs 9). Scale bar = 200 μm. (**B**) For the transwell migration or (**C**) invasion assay, cells were seeded in 24-well transwell plates and incubated for 18 h in medium containing 0.5% FBS. For the invasion assay, the invasion rate was measured using transwell plates in which the upper chamber was filled with matrigel. Cells were stained with crystal violet dye (top) and counted using a light microscope (bottom). Error bars represent means ± SD of all experiments. *p* values (bar vs bar, B-left panel) : *p* = 0.0008 (1 vs 2), *p* = 0.02 (1 vs 3). *p* values (bar vs bar, B-right panel) : *p* = 0.0002 (1 vs 2), *p* = 0.006 (1 vs 3). *p* values (bar vs bar, C-left panel) : *p* = 0.0001 (1 vs 2), *p* = 0.0004 (1 vs 3). *p* values (bar vs bar, C-right panel) : *p* = 0.0004 (1 vs 2), *p* = 0.004 (1 vs 3). Scale bar = 200 μm.

Based on previous work demonstrating that galectin-1 can activate cell migration and invasion in various cancer cell lines [44, 45], we investigated the effect of galectin-1 knock-down on the migration and invasion of human breast cancer cells. Figure 2A and 2B indicate that ablation of galectin-1 resulted in significant inhibition of cell migration of galectin-1 knock-down cells compared to control cells. The healed rate of the wounded area was reduced by 50% and 40% in MDA-MB-231 galectin-1 shRNA cells and Hs578T galectin-1 shRNA cells respectively, compared to each control shRNA cells (Figure 2A). The ability to migrate through the transwell was also decreased in galectin-1 knock-down cells by more than 50% (Figure 2B). In addition, we could observe significant reduction of cell invasion (90% in MDA-MB-231 and 50% in Hs578T) galectin-1 knock-down cells compared to each control shRNA cells (Figure 2C).

### Galectin-1 silencing promotes doxorubicin-induced apoptosis in human breast cancer cells

**Figure 3:**
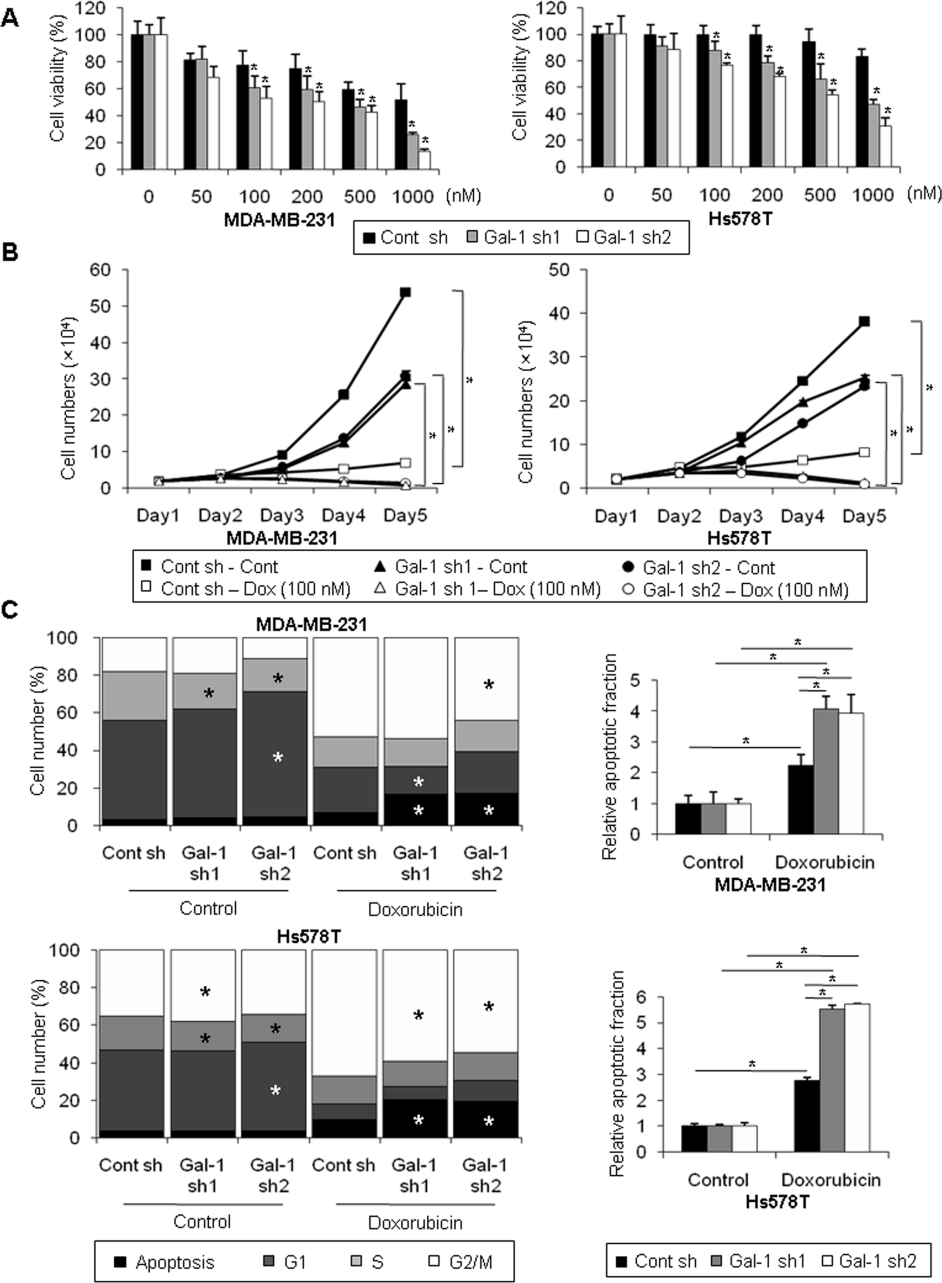
Silencing of galectin-1 decreases doxorubicin resistance of human breast cancer cells (**A**) Cells were seeded at a density of 3 × 10^3^ cells/well in 96-well plates and treated with the indicated concentration of doxorubicin for 48 h. Viability was then assessed by the MTT assay. The results were expressed as the mean ± SD. *p* values (bar vs bar, left panel) : *p* = 0.004 (7 vs 8), *p* = 0.002 (7 vs 9), *p* = 0.009 (10 vs 11), *p* = 0.0001 (10 vs 12), *p* = 0.0004 (13 vs 14), *p* = 0.0006 (13 vs 15), *p* = 2.6E-05 (16 vs 17), *p* = 7.44E-08 (16 vs 18). *p* values (bar vs bar, right panel) : *p* = 0.005 (7 vs 8), *p* = 6.28E-07 (7 vs 9), *p* = 4.22E-06 (10 vs 11), *p* = 2.25E-09 (10 vs 12), *p* = 0.0001 (13 vs 14), *p* = 0.0001 (13 vs 15), *p* = 2.52E-10 (16 vs 17), *p* = 8.12E-13 (16 vs 18). (**B**) Proliferation of galectin-1 knock-down (MDA-MB-231, Gal-1 sh1 and Gal-1 sh2; Hs578T Gal-1 sh1 and Gal-1 sh2) cells and control (MDA-MB-231 Cont sh and Hs578T Cont sh) cells upon treatment with 0.1 μM of doxorubicin. Cells were seeded at a density of 2×10^4^ cells/well in 12-well plates and counted with a hemocytometer for 5 days. Medium containing fresh doxorubicin was replenished every 2 days. The results were expressed as the mean ± SD. *p* values (control vs doxorubicin, left panel) : *p* = 1.98E-08 (Cont sh), *p* = 1.98E-08 (Gal-1 sh1), *p* = 3.48E-06 (Gal-1 sh2). *p* values (control vs doxorubicin, right panel) : *p* = 5.7E-07 (Cont sh), *p* = 1.12E-07 (Gal-1 sh1), *p* = 5.70E-07 (Gal-1 sh2) (**C**) Cell cycle analysis of galectin-1 knock-down (MDA-MB-231, Gal-1 sh1 and Gal-1 sh2; Hs578T, Gal-1 sh1 and Gal-1 sh2) cells and control (MDA-MB-231 Cont sh and Hs578T Cont sh) cells upon treatment with doxorubicin. Cells were seeded at a density of 1 × 10^6^ cells in 100-mm dishes. Cells were treated with 0.5 μM doxorubicin for 48 h, fixed in methanol, and incubated in PBS containing 50 μg/ml propidium iodide (PI) and 1 mg/ml RNase. Cell cycle analysis was performed by flow cytometry. The results were expressed as the mean ± SD. *p* values (upper-left panel) : *p* = 0.006 (control, Cont sh vs Gal-1 sh1, S phase), *p* = 0.003 (control, Cont sh vs Gal-1 sh2, S phase), *p* = 0.004 (control, Cont sh vs Gal-1 sh2, G1 phase), *p* = 0.004 (doxorubicin, Cont sh vs Gal-1 sh1, G1 phase), *p* = 0.001 (doxorubicin, Cont sh vs Gal-1 sh1, G0 phase), *p* = 0.03 (doxorubicin, Cont sh vs Gal-1 sh2, G2/M phase), *p* = 0.004 (doxorubicin, Cont sh vs Gal-1 sh2, G0 phase). *p* values (bar vs bar, upper-right panel) : *p* = 0.009 (1 vs 4), *p* = 0.0007 (2 vs 5), *p* = 0.001 (3 vs 6), *p* = 0.004 (4 vs 5), *p* = 0.01 (4 vs 6). *p* values (lower-left panel) : *p* = 0.03 (control, Cont sh vs Gal-1 sh1, G2/M phase), *p* = 0.008 (control, Cont sh vs Gal-1 sh1, S phase), *p* = 0.004 (control, Cont sh vs Gal-1 sh2, S phase), *p* = 0.01 (control, Cont sh vs Gal-1 sh2, G1 phase), *p* = 9.39E-05 (doxorubicin, Cont sh vs Gal-1 sh1, G2/M phase), *p* = 1.5E-05 (doxorubicin, Cont sh vs Gal-1 sh1, G0 phase), *p* = 2.5E-05 (doxorubicin, Cont sh vs Gal-1 sh2, G2/M phase), *p* = 2.3E-06 (doxorubicin, Cont sh vs Gal-1 sh2, G0 phase). *p* values (bar vs bar, upper-right panel) : *p* = 3.04E-0.5 (1 vs 4), *p* = 1.58E-06 (2 vs 5), *p* = 5.66E-07 (3 vs 6), *p* = 2.08E-05 (4 vs 5), *p* = 2.01E-06 (4 vs 6).

To determine the effect of galectin-1 silencing on the drug sensitivity of human breast cancer cells, we induced apoptosis by treatment with doxorubicin, which damages cellular DNA. The results shown in Figure 3A and 3B indicate that treatment of doxorubicin resulted in massive cell death of galectin-1 knock-down cells in a dose-dependent and time-dependent manner, whereas cell death in control shRNA cells was relatively attenuated compared with galectin-1 knock-down cells. After doxorubicin treatment for 72 h, growth was inhibited by approximately 98% in galectin-1 knock-down cells, whereas growth was inhibited by only 79% in control cells (Figure 3B). To further confirm the effect of galectin-1 ablation on drug sensitivity, we analyzed the cell cycle by flow cytometry. We found that the sub-G1 apoptotic fraction was significantly higher in galectin-1 knock-down cells after doxorubicin treatment than in control shRNA cells (Figure 3C, Supplementary Figure 2). These results suggest that silencing of galectin-1 enhanced drug-induced cell death by increasing apoptosis.

### Silencing of galectin-1 enhances sensitivity to doxorubicin by down-regulating survivin expression

**Figure 4:**
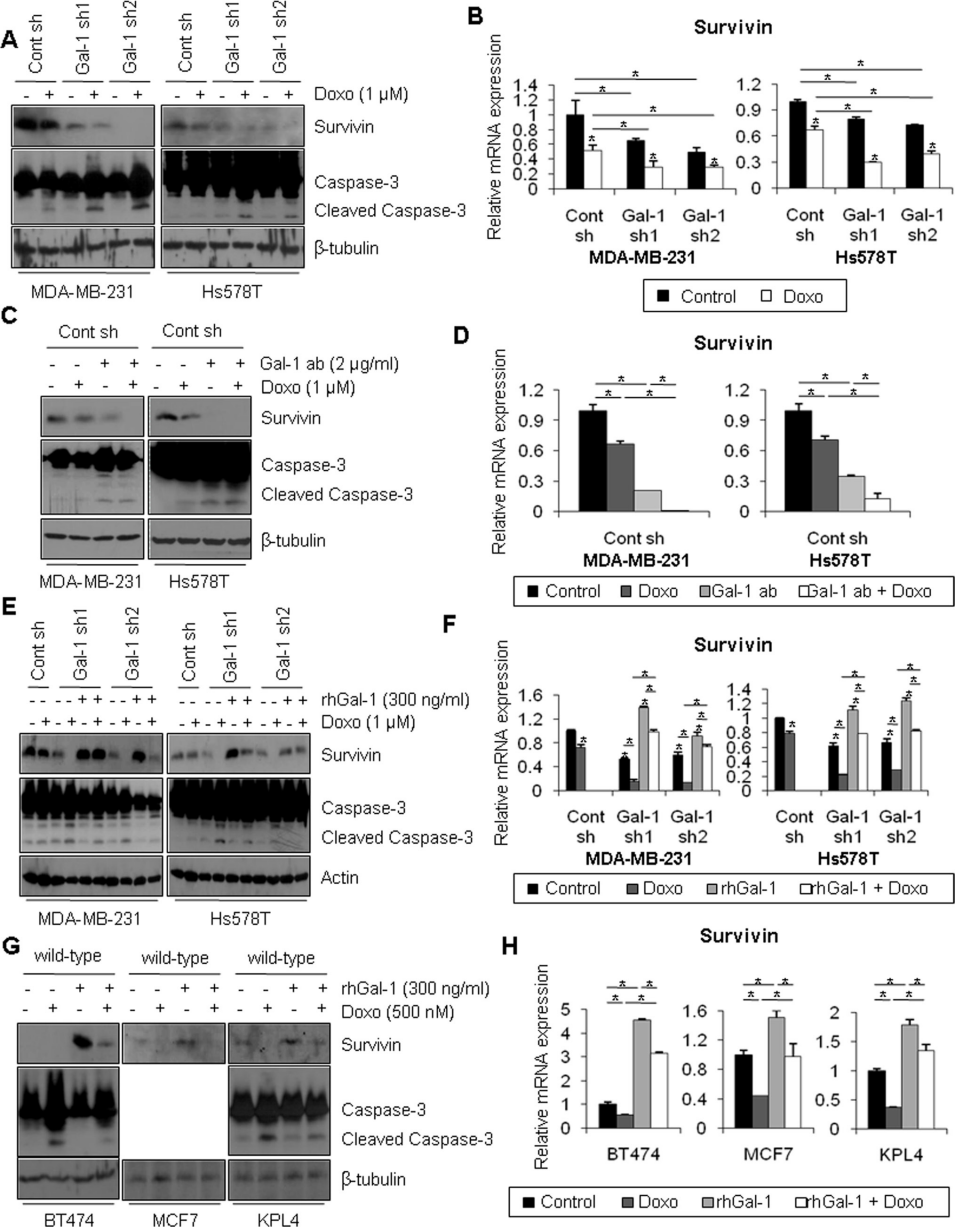
Ablation of galectin-1 blocks doxorubicin-induced survivin expression (**A**) Galectin-1 knock-down (MDA-MB-231, Gal-1 sh1 and Gal-1 sh2; Hs578T, Gal-1 sh1 and Gal-1 sh2) cells and control (MDA-MB-231 Cont sh and Hs578T Cont sh) cells were treated with 1 μM doxorubicin for 24 h. Cells were then harvested and analyzed by western blotting using antibodies specific for survivin, caspase-3, and β-tubulin. (**B**) qRT-PCR analysis of survivin mRNA levels in control shRNA and galectin-1 shRNA cells. qRT-PCR values were normalized to that of GAPDH mRNA. The results were expressed as the mean ± SD. *p* values (bar vs bar, left panel) : *p* = 0.02 (1 vs 2), *p* = 0.002 (3 vs 4), *p* = 0.007 (5 vs 6), *p* = 0.04 (1 vs 3), *p* = 0.01 (1 vs 5), *p* = 0.03 (2 vs 4), *p* = 0.01 (2 vs 6). *p* values (bar vs bar, right panel) : *p* = 0.005 (1 vs 2), *p* = 4.74E-06 (3 vs 4), *p* = 9.02E-05 (5 vs 6), *p* = 0.0004 (1 vs 3), *p* = 1.42E-05 (1 vs 5), *p* = 0.0002 (2 vs 4), *p* = 0.002 (2 vs 6). (**C**) At 24 h after cell seeding, control shRNA (MDA-MB-231 Cont sh and Hs578T Cont sh) cells were treated with doxorubicin (1 μM) and/or anti-galectin-1 antibodies (Gal-1 ab; 2 μg/ml). After 48 h, cell lysates were analyzed by western blotting and (**D**) qRT-PCR. qRT-PCR values were normalized to that of GAPDH mRNA. The results were expressed as the mean ± SD. *p* values (bar vs bar, left panel) : *p* = 0.0001 (1 vs 2), *p* = 8.43E-05 (2 vs 4), *p* = 0.002 (1 vs 3), *p* = 2.03E-05 (3 vs 4). *p* values (bar vs bar, right panel) : *p* = 0.0002 (1 vs 2), *p* = 6.26E-05 (2 vs 4), *p* = 0.0001 (1 vs 3), *p* = 0.002 (3 vs 4). (**E**) Galectin-1 knock-down (MDA-MB-231, Gal-1 sh1 and Gal-1 sh2; Hs578T, Gal-1 sh1 and Gal-1 sh2) cells were treated with doxorubicin (1 μM) and/or 300 ng/ml recombinant human galectin-1 (rhGal-1) and then incubated for 48 h. Cell lysates were then analyzed by western blotting and (**F**) qRT-PCR. qRT-PCR values were normalized to that of GAPDH mRNA. Error bars represent means ± SD of all experiments. *p* values (bar vs bar, left panel) : *p* = 0.0003 (1 vs 2), *p* = 0.0001 (1 vs 3), *p* = 4.24E-05 (3 vs 4), *p* = 0.0002 (3 vs 5), *p* = 2.25E-05 (5 vs 6), *p* = 5.22E-05 (4 vs 6), *p* = 2.02E-05 (1 vs 7), *p* = 5.26E-05 (7 vs 8), *p* = 0.005 (7 vs 9), *p* = 0.03 (9 vs 10), *p* = 0.0006 (8 vs 10). *p* values (bar vs bar, right panel) : *p* = 0.0002 (1 vs 2), *p* = 6.78E-05 (1 vs 3), *p* = 5.24E-05 (3 vs 4), *p* = 0.0002 (3 vs 5), *p* = 2.03E-05 (5 vs 6), *p* = 3.4E-06 (4 vs 6), *p* = 0.0003 (1 vs 7), *p* = 0.0002 (7 vs 8), *p* = 9.69E-05 (7 vs 9), *p* = 6.39E-06 (9 vs 10), *p* = 0.0002 (8 vs 10). (**G**) At 24 h after seeding, BT474, MCF7, and KPL4 wild-type cells were treated with doxorubicin (0.5 μM) and/or recombinant human galectin-1 (rhGal-1; 300 ng/ml) for 48 h. Cell lysates were then analyzed by western blotting with the indicated antibodies and (**H**) qRT-PCR. qRT-PCR values were normalized to that of GAPDH mRNA. The results were expressed as the mean ± SD. *p* values (bar vs bar, BT474) : *p* = 0.002 (1 vs 2), *p* = 6.43E-05 (2 vs 4), *p* = 0.002 (1 vs 3), *p* = 0.001 (3 vs 4). *p* values (bar vs bar, MCF7) : *p* = 7.44E-05 (1 vs 2), *p* = 0.007 (2 vs 4), *p* = 0.001 (1 vs 3), *p* = 0.01 (3 vs 4). *p* values (bar vs bar, KPL4) : *p* = 7.12E-06 (1 vs 2), *p* = 0.0001 (2 vs 4), *p* = 0.0002 (1 vs 3), *p* = 0.006 (3 vs 4).

Survivin is a well-known member of the IAP (inhibitor of apoptosis protein) family, whose members can disrupt apoptotic signaling pathways, resulting in cell survival [46]. Motivated by the recent finding that survivin expression is up-regulated in TNBC cells [47], we analyzed survivin expression in breast cancer tissue specimens by western blotting. We found that survivin expression was significantly elevated in patients with TNBC compared with patients with non-TNBC breast cancer (Figure 1F). However, in normal breast tissues, we could not observe survivin expression (Supplementary Figure 1). To investigate whether the increase of doxorubicin-induced apoptosis in galectin-1 knock-down cells was due to the change in survivin expression, cells treated with doxorubicin were analyzed by western blotting and qRT-PCR. Treatment with doxorubicin resulted in significant reduction of survivin expression in galectin-1 knock-down cells, whereas survivin expression was suppressed to a lesser extent in control cells (Figure 4A, 4B).

Galectin-1 is a secreted protein that binds to carbohydrate-rich regions of cell surface proteins [4, 5]. To test whether the extracellular domain of galectin-1 affects survivin expression, control cells were subjected to co-treatment with doxorubicin and anti-galectin-1 antibodies, which can neutralize extracellular galectin-1. Interestingly, when cells were co-treated with doxorubicin and anti-galectin-1 antibodies, the presence of the anti-galectin-1 antibodies accelerated the suppression of survivin expression and induction of caspase-3 activity (Figure 4C, 4D). Moreover, when cells were co-treated with doxorubicin and recombinant human galectin-1, survivin expression was rescued, and the increased caspase-3 activity was attenuated by recombinant human galectin-1 in galectin-1 knock-down cells (Figure 4E, 4F). To confirm that extracellular galectin-1 up-regulates survivin expression, BT474, MCF7, and KPL4 cells (which express low levels of galectin-1) were co-treated with doxorubicin and recombinant human galectin-1 (Figure 4G, 4H). We found that treatment with recombinant human galectin-1 induced expression of survivin mRNA, up-regulated survivin protein level, and potentiated doxorubicin resistance (MCF7 cells do not express caspase-3). These results suggest that ablation of galectin-1 attenuates doxorubicin resistance via down-regulation of survivin expression.

### Galectin-1-induced chemoresistance by survivin is mediated by the FAK/c-Src/ERK/STAT3 signaling pathway

**Figure 5:**
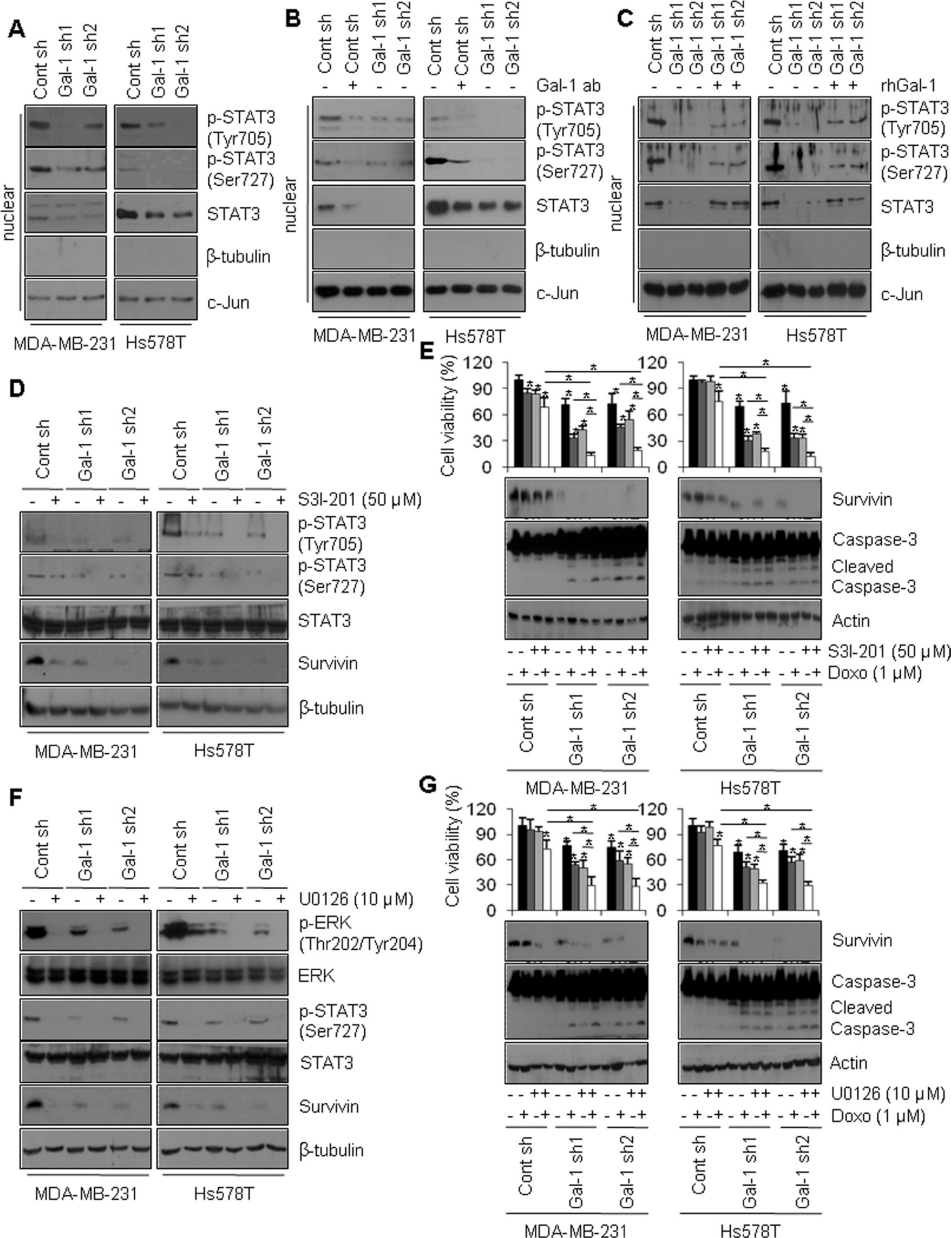
Galectin-1 drives survivin expression through the ERK/STAT3 pathway in human breast cancer cells (**A**) Effect of STAT3 signaling on survivin expression. Cells were seeded in a 100-mm dish at a density of 1 × 10^6^ cells and subjected to subcellular fractionation. The nuclear fraction was analyzed by western blotting using antibodies against β-tubulin as a cytosolic marker and c-Jun as a nuclear marker. (**B**, **C**) Effect of galectin-1 on survivin expression. Control shRNA (MDA-MB-231 Cont sh and Hs578T Cont sh) cells were treated with anti-galectin-1 antibodies (Gal-1 ab; 2 μg/ml), and (C) recombinant human galectin-1 (rhGal-1; 300 ng/ml) for 48 h. Nuclear fractions were assessed by western blotting. (**D**) Galectin-1 knock-down (MDA-MB-231, Gal-1 sh1 and Gal-1 sh2; Hs578T, Gal-1 sh1 and Gal-1 sh2) cells and control (MDA-MB-231 Cont sh and Hs578T Cont sh) cells were treated with the STAT3 inhibitor S3I-201 (50 μM) and subsequently analyzed by western blotting. (**E**) Effect of STAT3 signaling on doxorubicin-induced apoptosis. Cells were treated with doxorubicin (1 μM) and/or S3I-201 (50 μM) for 24 h and subjected to MTT assays and western blot analysis with the indicated antibodies. Error bars represent means ± SD of all experiments. *p* values (bar vs bar, left panel) : *p* = 5.95E-05 (1 vs 2), *p* = 1.04E-05 (1 vs 3), *p* = 3.29E-06 (1 vs 4), *p* = 6.74E-07 (1 vs 5), *p* = 8.8E-09 (5 vs 6), *p* = 2.25E-07 (5 vs 7), *p* = 2.24E-10 (7 vs 8), *p* = 1.42E-07 (6 vs 8), *p* = 2.89E-05 (1 vs 9), *p* = 1.59E-05 (9 vs 10), *p* = 0.004 (9 vs 11), *p* = 1.36E-07 (11 vs 12), *p* = 1.01E-09 (4 vs 8), *p* = 3.4E-10 (10 vs 12), *p* = 3.76E-09 (4 vs 12). *p* values (bar vs bar, right panel) : *p* = 4.75E-05 (1 vs 4), *p* = 3.83E-09 (1 vs 5), *p* = 2.53E-09 (5 vs 6), *p* = 4.29E-09 (5 vs 7), *p* = 2.16E-09 (7 vs 8), *p* = 2.23E-05 (6 vs 8), *p* = 8.63E-05 (1 vs 9), *p* = 2.65E-06 (9 vs 10), *p* = 1.46E-06 (9 vs 11). *p* = 1.35E-07 (11 vs 12), *p* = 3.38E-09 (4 vs 8), *p* = 3.71E-07 (10 vs 12), *p* = 1.34E-09 (4 vs 12). (**F**) Control and galectin-1 knock-down cells were treated with U0126 (10 μM) for 24 h and subsequently analyzed by western blotting. (**G**) Effect of the ERK pathway on doxorubicin-induced apoptosis. Control and galectin-1 knock-down cells were treated with doxorubicin (1 μM) and/or U0126 (10 μM) for 24. MTT assays and western blotting were then performed. Error bars represent means ± SD of all experiments. *p* values (bar vs bar, left panel) : *p* = 6.22E-05 (1 vs 4), *p* = 1.8E-05 (1 vs 5), *p* = 7.92E-08 (5 vs 6), *p* = 9.23E-06 (5 vs 7), *p* = 0.001 (7 vs 8), *p* = 6.3E-06 (6 vs 8), *p* = 2.11E-05 (1 vs 9), *p* = 0.0005 (9 vs 10), *p* = 9.14E-05 (9 vs 11). *p* = 2.71E-05 (11 vs 12), *p* = 4.73E-07 (4 vs 8), *p* = 0.0004 (10 vs 12), *p* = 2.34E-07 (4 vs 12). *p* values (bar vs bar, right panel) : *p* = 2.94E-05 (1 vs 4), *p* = 2.08E-06 (1 vs 5), *p* = 0.0004 (5 vs 6), *p* = 8.5E-05 (5 vs 7), *p* = 8.1E-06 (7 vs 8), *p* = 8.1E-06 (6 vs 8), *p* = 3.04E-06 (1 vs 9), *p* = 0.001 (9 vs 10), *p* = 0.006 (9 vs 11). *p* = 4.89E-08 (11 vs 12), *p* = 1.48E-10 (4 vs 8), *p* = 2.51E-08 (10 vs 12), *p* = 1.18E-10 (4 vs 12).

Based on our finding that galectin-1 ablation inhibited survivin expression in human breast cancer cells, we investigated the mechanism by which galectin-1 promotes survivin expression. Previous reports have indicated that survivin expression is increased by various transcription factors, such as Sp1 [39], HIF-1α [40], Egr-1 [41], and STAT3 [35]. To determine the transcriptional regulator of survivin in our system, we performed cell fractionation experiments to determine the effect of galectin-1 silencing on the nuclear localization of candidate translocation factors that could potentially decrease survivin expression. As shown in Figure 5A and Supplementary Figure 3, only STAT3 nuclear translocation was blocked by galectin-1 silencing. We also observed reductions in nuclear translocation of Tyr705-phosphorylated and Ser727-phosphorylated STAT3 in galectin-1 knock-down cells. Phosphorylation of these two residues has been shown to be required for STAT3 nuclear translocation [26, 48]. In addition, consistent with results obtained in cells with stably silenced galectin-1-expression, control cells treated with anti-galectin-1 antibodies exhibited decreased level of STAT3 (Figure 5B). When the galectin-1 knock-down cells were treated with recombinant human galectin-1, nuclear translocation of Tyr705-and Ser727-phosphorylated STAT3 was restored to normal levels (Figure 5C). These results suggest that extracellular galectin-1 increases survivin expression by regulating STAT3 nuclear translocation. Since previous publications have reported that STAT3 is physically associated with the survivin promoter region (−1231/−1009) [35], we tested whether changes in survivin level by galectin-1 ablation might be mediated by STAT3. The results in Supplementary Figure 3B indicated that the association of STAT3 with the survivin promoter region was decreased in galectin-1 shRNA cells compared to control shRNA cells, as determined by chromatin immunoprecipitation assays. These results indicate that silencing of galectin-1 suppresses STAT3 binding to survivin promoter via reduction of STAT3 nuclear translocation.

To confirm the effect of STAT3 phosphorylation on survivin expression, cells were treated with S3I-201, a STAT3 inhibitor, to inhibit STAT3 phosphorylation and nuclear translocation [49]. Cells treated with S3I-201 exhibited decreased survivin mRNA and protein levels compared to non-treated control cells (Figure 5D, Supplementary Figure 3C). In addition, doxorubicin and S3I-201 synergistically reduced cell survival and survivin protein level in galectin-1 knock-down cells, whereas the magnitudes of their synergistic effects were reduced in control cells (Figure 5E). We also observed enhanced levels of caspase-3 cleavage in galectin-1 knock-down cells co-treated with doxorubicin and S3I-201 (Figure 5E).

Residue Ser727 on STAT3 has been reported to be phosphorylated by ERK [50]. When we treated cells with U0126, a MEK inhibitor, we observed decreased Ser727 phosphorylation in STAT3 and decreased survivin expression (Figure 5F, Supplementary Figure 3D). To further confirm the effect of the ERK pathway on DNA damage-induced apoptosis, we determined the effect of U0126 on doxorubicin-induced apoptosis. As shown in Figure 5G, galectin-1 knock-down cells co-treated with U0126 and doxorubicin exhibited significantly reduced survival and survivin level, whereas caspase-3 activity was enhanced. Less prominent decreases were observed in control cells (Figure 5G). Taken together, these observations suggest that galectin-1-dependent modulation of doxorubicin resistance is enhanced by survivin via ERK-induced STAT3 Ser727 phosphorylation, at least in the cell lines examined here.

**Figure 6:**
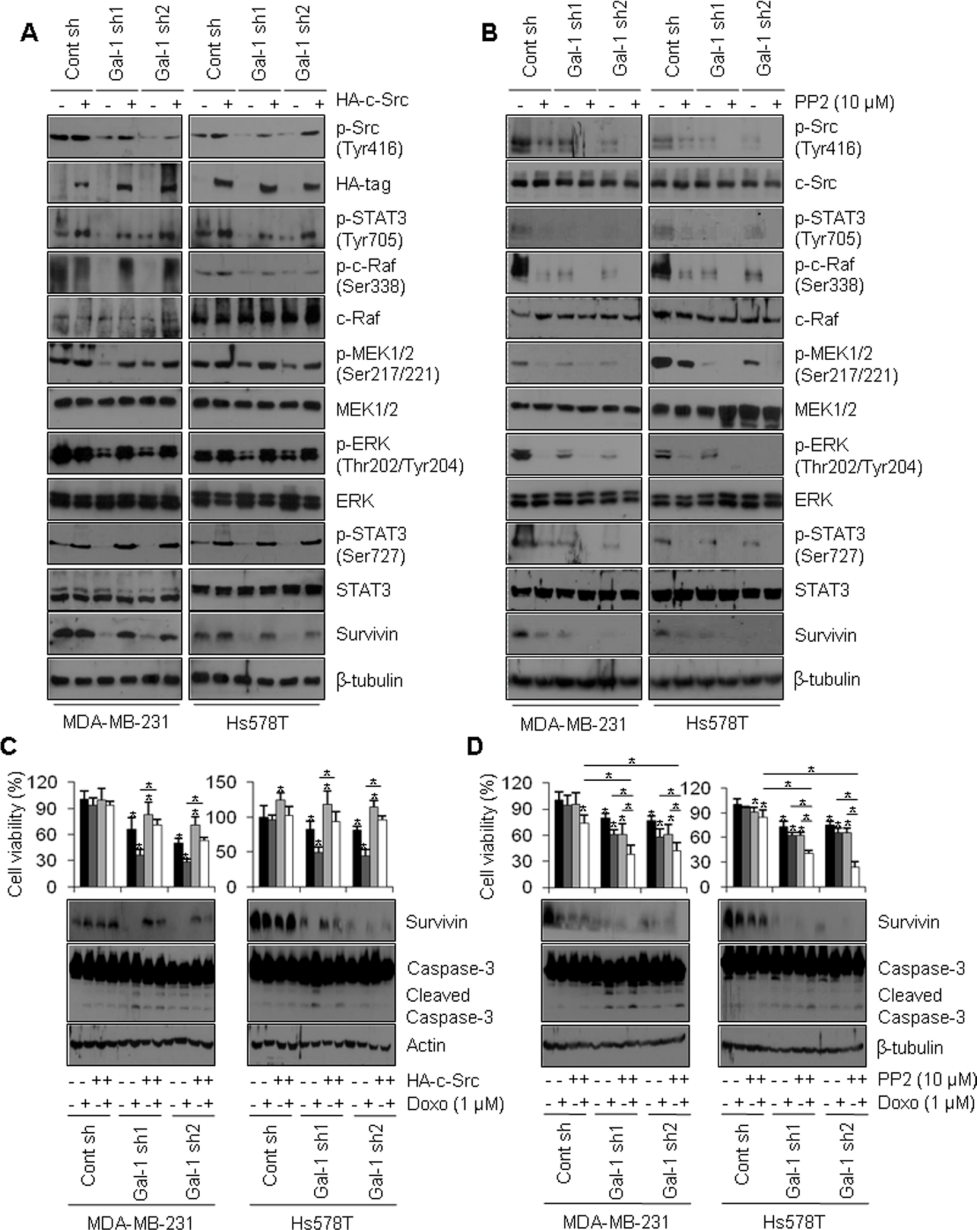
Galectin-1 ablation attenuates survivin expression via the c-Src/ERK/STAT3 signaling pathway (**A**) Cells were seeded at a density of 1 × 10^6^ cells in 100-mm dishes. After seeding, the cells were transfected with a construct driving the expression of HA-tagged c-Src or (**B**) treated with PP2 (10 μM) and subsequently analyzed by western blotting. (**C**, **D**) Effect of c-Src signaling on doxorubicin-induced apoptosis. Cells were seeded in a 100-mm dish at a density of 1 × 10^6^ cells. At 24 h after cell seeding, (C) cells were transfected with a construct driving the expression of HA-tagged c-Src, incubated for 48 h, and analyzed by the MTT assay and western blotting. Cells were then treated with doxorubicin (1 μM) for 24 h. Error bars represent means ± SD of all experiments. *p* values (bar vs bar, left panel) : *p* = 0.0008 (1 vs 5), *p* = 9.15E-05 (5 vs 6), *p* = 0.03 (5 vs 7), *p* = 2.57E-08 (6 vs 8), *p* = 2.93E-06 (1 vs 9), *p* = 2.15E-07 (9 vs 10), *p* = 0.0001 (9 vs 11), *p* = 1.23E-09 (10 vs 12). *p* values (bar vs bar, right panel) : *p* = 0.003 (1 vs 3), *p* = 0.03 (1 vs 5), *p* = 6.34E-06 (5 vs 6), *p* = 0.0002 (5 vs 7), *p* = 1.79E-06 (6 vs 8), *p* = 0.007 (1 vs 9), *p* = 1.65E-07 (9 vs 10), *p* = 7.69E-07 (9 vs 11), *p* = 7.27E-10 (10 vs 12). (D) Cells were treated with doxorubicin (0.5 μM) and/or PP2(10 μM) for 24 h, after which they were analyzed by the MTT assay and western blotting. Error bars represent means ± SD of all experiments. *p* values (bar vs bar, left panel) : *p* = 7.93E-05 (1 vs 4), *p* = 0.0001 (1 vs 5), *p* = 6.73E-06 (5 vs 6), *p* = 0.001 (5 vs 7), *p* = 0.002 (7 vs 8), *p* = 7.59E-05 (6 vs 8), *p* = = 3.03E-05 (1 vs 9), *p* = 0.0002 (9 vs 10), *p* = 0.0002 (9 vs 11), *p* = 0.03 (11 vs 12), *p* = 5.85E-06 (4 vs 8), *p* = 0.003 (10 vs 12), *p* = 1.36E-05 (4 vs 12). *p* values (bar vs bar, right panel) : *p* = 0.01 (1 vs 3), *p* = 0.002 (1 vs 4), *p* = 8.34E-07 (1 vs 5), *p* = 0.0007 (5 vs 6), *p* = 0.03 (5 vs 7), *p* = 0.0001 (7 vs 8), *p* = 7.61E-09 (6 vs 8), *p* = 4.58E-07 (1 vs 9), *p* = 0.002 (9 vs 10), *p* = 0.01 (9 vs 11). *p* = 4.89E-09 (11 vs 12), *p* = 6.76E-10 (4 vs 8), *p* = 8.43E-09 (10 vs 12), *p* = 4.45E-10 (4 vs 12).

Based on our finding that ERK phosphorylates STAT3 on Ser727, we investigated whether Tyr705 phosphorylation of STAT3 is stimulated by galectin-1. One previous study reported that Tyr705 phosphorylation of STAT3 is mediated by a tyrosine kinase, c-Src [29]. Moreover, c-Src is known to act as a central molecule that up-regulates the ERK pathway [51]. Therefore, we hypothesized that galectin-1-induced doxorubicin resistance via regulation of survivin expression might be mediated by c-Src-mediated phosphorylation of Tyr705 and the ERK-mediated phosphorylation of Ser727 in STAT3. To verify this in our cell lines, we transfected cells with HA-tagged c-Src or treated cells with the Src kinase inhibitor PP2 and examined the levels of downstream signaling molecules. Overexpression of HA-tagged c-Src increased the levels of phosphorylated c-Raf, MEK1/2, and ERK and also increased the phosphorylation of STAT3 on Tyr705 and Ser727. c-Src expression also increased survivin levels in our cell lines (Figure 6A, Supplementary Figure 3E). Accordingly, treatment with PP2 resulted in decreased c-Raf, MEK1/2, and ERK phosphorylation, decreased STAT3 Tyr705/Ser727 phosphorylation, and decreased expression of survivin in our cell lines (Figure 6B, Supplementary Figure 3F). In addition, in doxorubicin-treated galectin-1 knock-down cells, c-Src overexpression increased cell viability, increased survivin protein level, and decreased caspase-3 cleavage (Figure 6C). In contrast, in doxorubicin and PP2 co-treated galectin-1 knock-down cells, cell survival and survivin expression were attenuated, whereas caspase-3 cleavage was increased (Figure 6D). These results collectively suggest that ablation of galectin-1 decreases doxorubicin resistance via down-regulation of the c-Src/ERK/STAT3/survivin pathway.

### Galectin-1 binding to integrin β1 potentiates doxorubicin resistance by driving the FAK/c-Src/ERK/STAT3/survivin pathway

**Figure 7:**
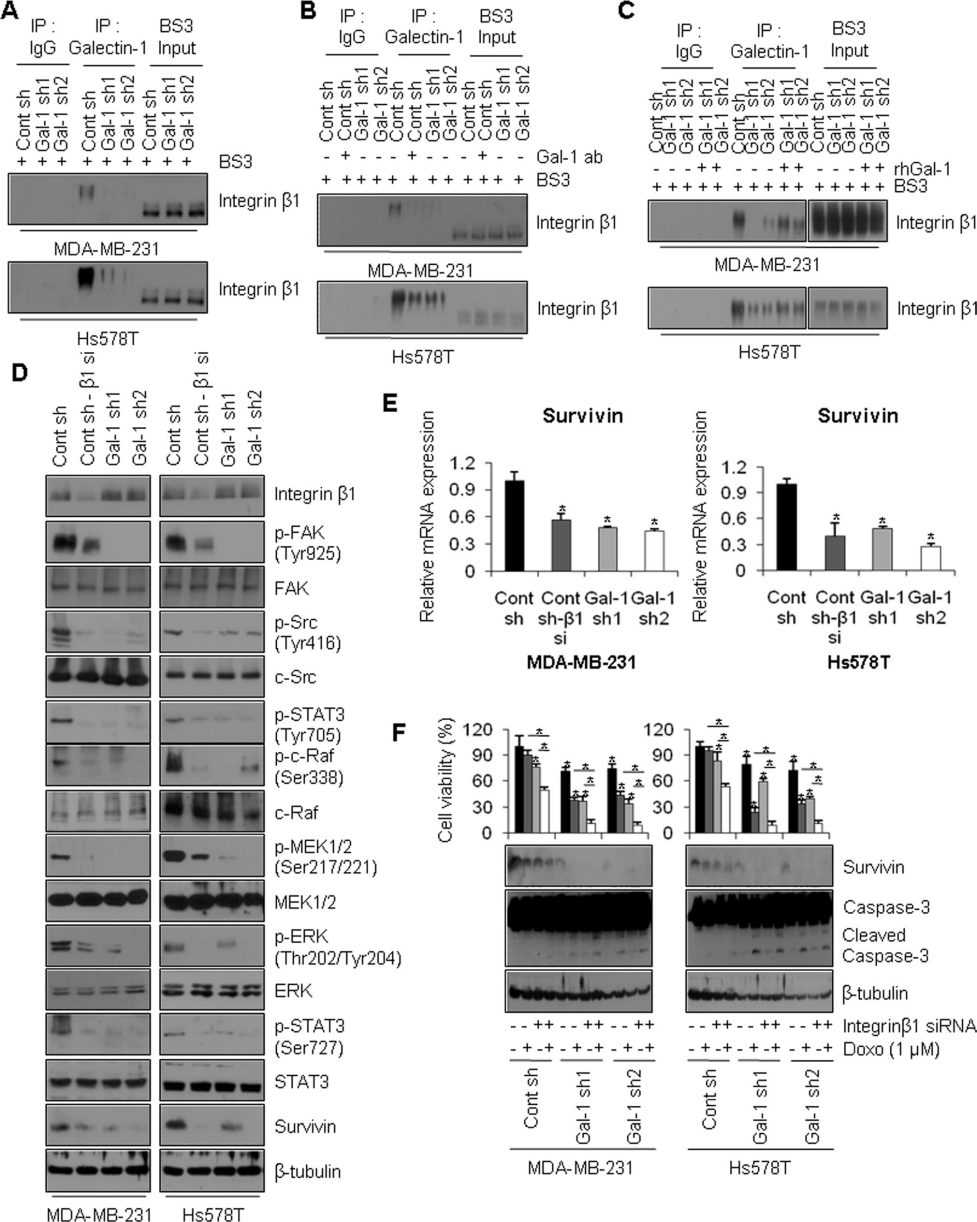
Extracellular galectin-1 up-regulates the FAK/c-Src/ERK/STAT3/survivin pathway by directly interacting with integrin β1 (**A**) Assessment of galectin-1 binding to integrin β1. Cells were seeded in 100-mm dishes at a density of 1 × 10^6^cells. Cells were then incubated with 1 mM BS^3^ for 20 min to cross-link galectin-1 to cell surface proteins. Lysates were immunoprecipitated with anti-galectin-1 antibody and analyzed by western blotting with anti-integrin β1 antibody. (**B**) At 24 h after cell seeding, control shRNA (MDA-MB-231 Cont sh and Hs578T Cont sh) cells were treated with anti-galectin-1 antibodies (Gal-1 ab; 2 μg/ml) and further incubated for 48 h. Treated cells were then incubated with BS^3^. Next, cell lysates were immunoprecipitated with anti-galectin-1 antibody and analyzed by western blotting with anti-integrin β1 antibody. (**C**) Cells were seeded in 100-mm dishes at a density of 1 × 10^6^ cells. Galectin-1 knock-down (MDA-MB-231, Gal-1 sh1 and Gal-1 sh2; Hs578T, Gal-1 sh1 and Gal-1 sh2) cells were then treated with recombinant human galectin-1 (rhGal-1; 300 ng/ml) for 48 h. Next, cell lysates were immunoprecipitated with anti-galectin-1 antibody and analyzed by western blotting with anti-integrin β1 antibody. (**D**) Effect of integrin β1 on the FAK/c-Src/ERK/STAT3/survivin pathway. Control shRNA (MDA-MB-231 Cont sh and Hs578T Cont sh) cells were transfected with integrin β1-targeting siRNA for 48 h and analyzed by western blotting and (**E**) qRT-PCR. qRT-PCR values were normalized to that of GAPDH mRNA. The results were expressed as the mean ± SD. *p* values (bar vs bar, left panel) : *p* = 0.003 (1 vs 2), *p* = 0.0008 (1 vs 3), *p* = 0.0007 (1 vs 4). *p* values (bar vs bar, right panel) : *p* = 0.003 (1 vs 2), *p* = 0.0002 (1 vs 3), *p* = 5.69E-05 (1 vs 4). (**F**) Effect of integrin β1 on doxorubicin-induced apoptosis. Cells were transfected with integrin β1-targeting siRNA for 48 h and then subjected to MTT assays and western blot analysis with the indicated antibodies. Cells were then treated with 1 μM doxorubicin for 24 h. Error bars represent means ± SD of all experiments. *p* values (bar vs bar, left panel) : *p* = 8.1E-05 (1 vs 3), *p* = 2.79E-10 (3 vs 4), *p* = 9.39E-11 (2 vs 4), *p* = 1.56E-05 (1 vs 5), *p* = 1.02E-10 (5 vs 6), *p* = 1.85E-09 (5 vs 7), *p* = 1.76E-07 (7 vs 8), *p* = 6.64E-09 (6 vs 8), *p* = 6.06E-05 (1 vs 9), *p* = 4.85E-09 (9 vs 10), *p* = 3.3E-10 (9 vs 11), *p* = 1.35E-08 (11 vs 12), *p* = 2.28E-11 (10 vs 12). *p* values (bar vs bar, right panel) : *p* = 0.001 (1 vs 3), *p* = 4.86E-06 (3 vs 4), *p* = 1.97E-11 (2 vs 4), *p* = 7.66E-05 (1 vs 5), *p* = 7.22E-10 (5 vs 6), *p* = 5.52E-05 (5 vs 7), *p* = 5.98E-13 (7 vs 8), *p* = 4.43E-05 (6 vs 8), *p* = 1.24E-05 (1 vs 9), *p* = 1.96E-07 (9 vs 10), *p* = 6.78E-07 (9 vs 11), *p* = 3.99E-11 (11 vs 12), *p* = 8.92E-09 (10 vs 12).

We previously demonstrated that galectin-1 enhances doxorubicin resistance via modulation of the c-Src/ERK/STAT3/survivin pathway. Galectin-1 is present in both intracellular and extracellular space and the functions of secreted galectin-1 are mediated by its binding to various cell surface proteins such as integrin β1 [7]. This interaction activates cytoskeletal-associated proteins, FAK, and downstream c-Src/ERK pathways. To verify this interaction in our cell lines, we investigated whether galectin-1 binds directly to integrin β1 on the cell surface using a cross-linking reagent, BS^3^. As shown in Figure 7A, galectin-1 binds directly to integrin β1 on the cell surface. Moreover, ablation of galectin-1 decreased its binding to integrin β1. Interestingly, galectin-1-integrin β1 cross-linked bands migrated at an apparent molecular weight 30 kDa larger than that of integrin β1 bands in input samples. These results indicate that the dimeric form of galectin-1 (28 kDa) was bound to integrin β1.

Based on these results, we treated cells with anti-galectin-1 antibodies to neutralize the actions of extracellular galectin-1. Alternatively, we treated cells with recombinant human galectin-1 to mimic the effects of secreted galectin-1. We observed inhibition of galectin-1-integrin β1 binding in control cells treated with anti-galectin-1 antibodies (lane 6, Figure 7B). We also observed induction of galectin-1-integrin β1 binding in galectin-1 knock-down cells treated with recombinant human galectin-1 (lanes 9, 10, Figure 7C). To confirm the role of integrin β1-galectin-1 binding in survivin-mediated doxorubicin resistance, MDA-MB-231 cells and Hs578T control cells were transfected with siRNA targeting integrin β1. Upon siRNA-mediated silencing of integrin β1, downstream FAK/c-Src activities were decreased. These effects were followed by reduced c-Raf, MEK1/2, ERK, and STAT3 phosphorylation and reduced survivin expression on both the protein and mRNA levels (Figure 7D, 7E). Also, treatment with doxorubicin resulted in massive cell death, decreased survivin expression, and greater caspase-3 cleavage in integrin β1 knock-down cells compared to non-transfected control cells (Figure 7F). These results suggest that galectin-1 inhibits drug-induced apoptotic cell death by directly binding to cell surface integrin β1, thereby modulating the downstream FAK/c-Src/ERK/STAT3/survivin pathway.

### Rescue of galectin-1 ablation recovers doxorubicin resistance via restoration of the FAK/c-Src/ERK/STAT3/survivin pathway

**Figure 8:**
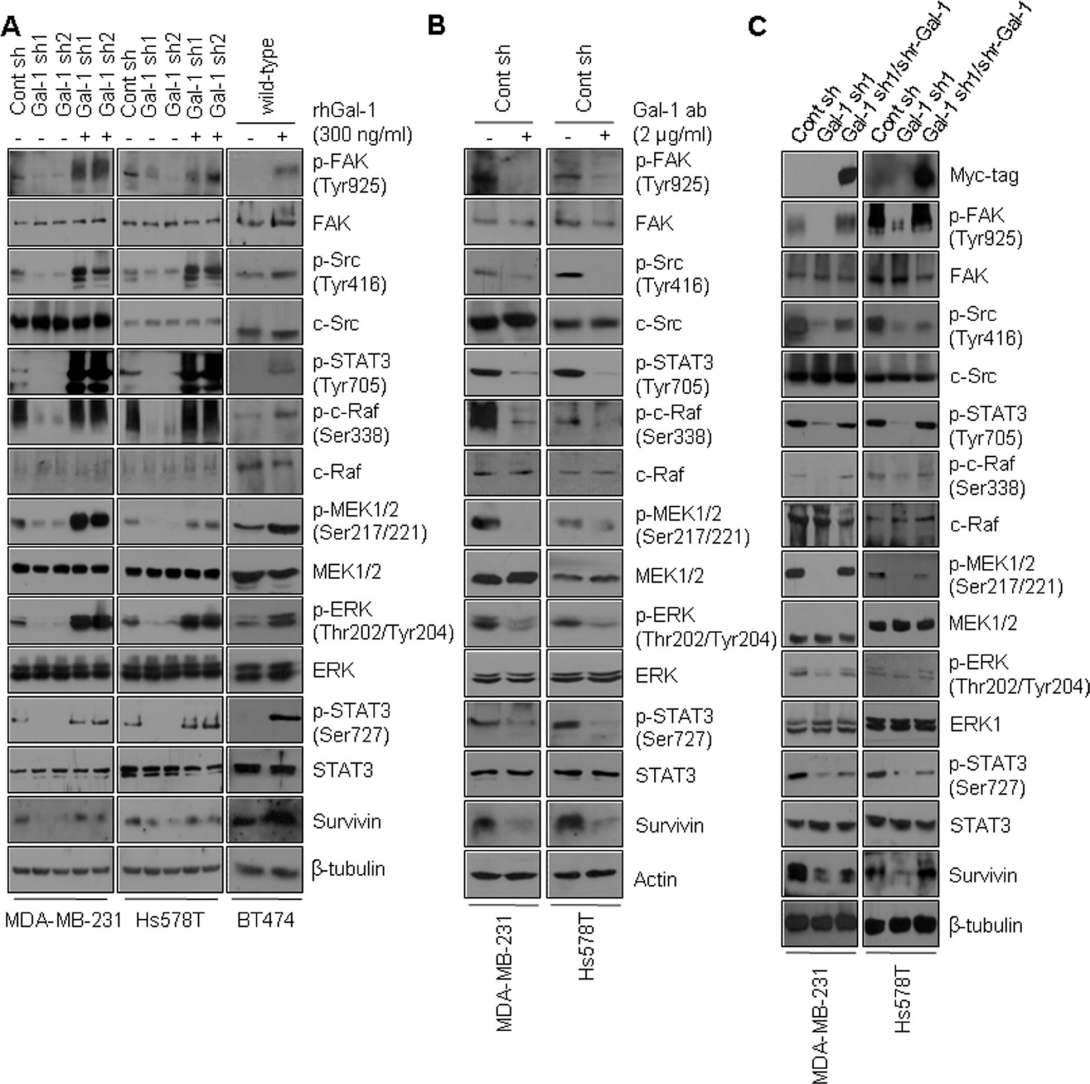
Rescue of galectin-1 knock-down restores FAK/c-Src/ERK/STAT3/survivin signaling (**A**) Effect of galectin-1 on the FAK/c-Src/ERK/STAT3/survivin pathway. Cells were seeded in 100-mm dishes at a density of 1 × 10^6^ cells. Galectin-1 knock-down (MDA-MB-231, Gal-1 sh1 and Gal-1 sh2; Hs578T, Gal-1 sh1 and Gal-1 sh2) cells and BT474 wild-type cells were treated with recombinant human galectin-1 (rhGal-1; 300 ng/ml) for 48 h and analyzed by western blotting. (**B**) At 24 h after cell seeding, control shRNA (MDA-MB-231 Cont sh and Hs578T Cont sh) cells were treated with anti-galectin-1 antibodies (Gal-1 ab; 2 μg/ml) and incubated for a further 48 h. The cells were then analyzed by western blotting with the indicated antibodies. (**C**) The pBabe-puro-myc/his-shRNA resistant galectin-1 construct was transfected into MDA-MB-231 Gal-1 sh1 and Hs578T Gal-1 sh1 cells. Cell lysates were then subjected to western blot analysis.

Based on our finding that galectin-1 down-regulates doxorubicin-induced apoptosis by directly binding to integrin β1, thereby activating the downstream FAK/c-Src/ERK/STAT3/survivin pathway, we next examined whether galectin-1-mediated chemoresistance is mediated by the FAK/c-Src/ERK/STAT3/survivin pathway. Recombinant human galectin-1was prepared (Supplementary Figure 4A) and used to treat galectin-1 knock-down cells and BT474, MCF7, and KPL4 wild-type cells, which exhibit low levels of galectin-1. Upon treatment with recombinant human galectin-1, the galectin-1 and integrin β1 interaction was increased, phosphorylation of FAK, c-Src, c-Raf, MEK1/2, and ERK was increased and STAT3 activity was also increased (Figure 8A, Supplementary Figure 4B). Survivin mRNA and protein expression levels were also increased by recombinant galectin-1 in our cell lines (Supplementary Figure 4C). In contrast, treatment with anti-galectin-1 antibodies inhibited FAK, c-Src, c-Raf, MEK1/2, ERK, and STAT3 phosphorylation. This treatment also down-regulated survivin levels in our cell lines (Figure 8B).

To further assess the effect of galectin-1 on FAK/c-Src/ERK/STAT3 activity and survivin expression, we reintroduced a shRNA-resistant version of the pSuper galectin-1 construct into MDA-MB-231 cells. We also introduced Hs578T pSuper galectin-1 shRNA clone 1 into cells (Figure 8C, Supplementary Figure 4D). We found that reintroduction of galectin-1 restored phosphorylation of FAK, c-Src, c-Raf, MEK1/2, ERK, and STAT3 in galectin-1 knock-down cells. The results shown in Figure 8 collectively suggest that silencing of galectin-1 efficiently decreases integrin β1 downstream signaling.

## DISCUSSION

Among the various breast cancer subtypes, TNBC is distinguished by its highly aggressive phenotype and poor prognostic features [2, 3]. Hence, intense attention has focused on finding therapeutics for TNBC. Since TNBC cells lack the HER2 cell surface receptor and nuclear hormone receptors, targeted therapeutics such as herceptin (which targets HER2) and estrogen receptor-targeting drugs are not suitable [42, 43]. Therefore, much effort has been spent in the identification of a specific therapeutic marker that can be used as a TNBC target molecule. As part of this effort, we focused on galectin-1. This focus was motivated by the recent finding that galectin-1 expression is significantly up-regulated in TNBC cells [52]. We established galectin-1 knock-down cell lines using two TNBC cell lines that endogenously express galectin-1, MDA-MB-231 and Hs578T, and determined the effects of galectin-1 silencing in these cell lines. To date, many studies have indicated that intracellular galectin-1 promotes cancer progression [44–46, 48, 51]. Based on our finding that galectin-1 is effectively secreted from TNBC cells, we focused on the extracellular action of secreted galectin-1 on TNBC cells. We found that extracellular galectin-1 suppressed doxorubicin-induced apoptosis by regulating integrin β1 downstream signaling, suggesting that galectin-1 is a novel molecular therapeutic marker.

We also found that galectin-1 was overexpressed in TNBC cell lines compared to non-TNBC cell lines. In addition, galectin-1 expression was significantly up-regulated in patients with TNBC compared with patients with non-TNBC. Moreover, we confirmed the binding of galectin-1 to the cell surface via its interaction with integrin β1, indicating that galectin-1 is a potential TNBC-specific cell surface marker. Using a combination of galectin-1 silencing, treatment with anti-galectin-1 antibodies, and treatment with recombinant galectin-1, we found that galectin-1 plays a seminal role in TNBC cells by regulating FAK/c-Src/ERK/STAT3 signaling.

Survivin, a member of the IAP family [38, 47], is a well-known cell cycle regulatory protein that interacts with mitotic spindle microtubules during mitosis [53]. Previous study has reported that survivin down-regulates the activity of caspase-3/7/9 and survivin overexpression enhances resistance to apoptosis induced by cell death signaling [54]. We found that survivin was overexpressed in TNBC cell lines compared to non-TNBC cell lines, and that ablation of galectin-1 attenuated doxorubicin-induced survivin expression, leading to enhanced apoptotic cell death. We conclude that the increase in doxorubicin-induced apoptotic cell death in galectin-1-silenced cells is due to suppressed survivin level.

STAT3 is thought to drive the transcription of various genes that up-regulate angiogenesis, cell cycle progression, and cell survival [30–35]. Among these genes, survivin gene is reported to contain STAT3 binding sites in promoter region and STAT3 activation correlates with expression of survivin [35]. For STAT3 activation, EGFR and/or c-Src-induced phosphorylation of Tyr705 and ERK-mediated phosphorylation of Ser727 are both required [27, 29, 50]. In our study, we showed a decrease in nuclear translocation of STAT3 in galectin-1 knock-down cells and inhibition of STAT3 nuclear translocation resulted in suppression of survivin expression. We also observed that pharmacological inhibition of MEK or c-Src activity decreased survivin expression. These results suggest that galectin-1-mediated modulation of survivin expression results from activated c-Src and ERK signaling, leading to phosphorylation of STAT3 on Tyr705 and Ser727 and subsequent translocation of STAT3 to the nucleus, where it drives the transcription of survivin.

Galectin-1 is a commonly secreted protein that interacts with various extracellular matrix proteins [4, 5]. Integrins are glycoproteins with galactosyl residues and β1 subunit of integrin was reported to directly interact with galectin-1 [7]. In agreement with previous studies, we confirmed that galectin-1 binds to integrin β1. We also found that the FAK/c-Src/ERK/STAT3/survivin pathway, which is downstream of integrin β1, is inhibited by galectin-1 silencing. In addition, after treatment with doxorubicin, cell viability and survivin expression were further decreased in integrin β1-knock-down cells compared to control cells. This finding suggests that integrin β1 mediates galectin-1 signaling.

In conclusion, our findings indicate that galectin-1 plays a pivotal role in the regulation of key processes in cancer cells, such as migration, invasion, and chemoresistance, by modulating FAK and ERK signaling and survivin level. We propose that silencing of galectin-1 attenuates survivin expression by decreasing the interaction of survivin with integrin β1. This decreases FAK and c-Src phosphorylation, which in turn inhibits phosphorylation of STAT3 on Tyr705. As a result of the decrease in c-Src phosphorylation, downstream ERK signaling is attenuated, and ERK-induced STAT3 Ser727 phosphorylation is decreased. Since STAT3 activity is suppressed, STAT3 does not dimerize or translocate to the nucleus, resulting in decreased survivin expression. We propose that cell surface-bound galectin-1, which up-regulates intracellular signaling culminating in survivin expression and resistance to doxorubicin, is a promising TNBC-specific therapeutic target (Supplementary Figure 5).

## MATERIALS AND METHODS

### Plasmids

shRNA constructs targeting different sequences of human galectin-1 and scrambled shRNA constructs were kindly donated by Dr. Gabriel A. Rabinovich (Buenos Aires University, Buenos Aires, Argentina). Constructs driving the expression of FLAG-tagged galectin-1 and HA-tagged c-Src were kindly provided by Dr. Kuo-I Lin (National Taiwan University, Taipei, Taiwan) and Dr. Wook Jin (Gachon University, Incheon, Korea). Using the galectin-1-WT construct as a backbone, an shRNA-resistant galectin-1 construct harboring a wobble mutation was produced by a two-step PCR procedure employing the overlap extension PCR method. Briefly, in the first step, two PCR reactions were performed. One reaction was performed with primers 1 and 2; the other reaction was performed with primers 3 and 4. Amplifications were performed using a Pfu DNA polymerase kit (Elpis Biotech, Daejeon, Korea). The two products from the first set of PCR reactions were mixed in equimolar concentrations and used as templates for the second PCR reaction, which utilized primers 1 and 4. The resulting product was subcloned into pBabe-puro using the BamH1 and Sal1 restriction sites. Primer sequences were as follows: Primer 1: 5′-CTA GGA TCC ATG GCT TGT GGT CTG GTC GCC AGC AAC-3′, Primer 2: 5′-CTC GTA GCC GTC AGG GAG CTT GAC GGT CAG GTT G-3′, Primer 3: 5′-GCT CCC TGA CGG CTA CGA GTT CAA GTT CCC CAA C-3′, and Primer 4: 5′-CTA GTC GAC TCA GTC AAA GGC CAC ACA TTT GAT CTT G-3′.

### Cell culture and transfection

MDA-MB-231, Hs578T, BT474, T47D, MCF7, and 293T cells were maintained in Dulbecco's modified Eagle's medium (DMEM) supplemented with 10% fetal bovine serum (FBS), 100 U/ml penicillin, and 100 mg/ml streptomycin. Cells were incubated at 37°C in a humidified atmosphere with 5% CO_2_. Calcium phosphate transfection was performed in 293T cells as described previously [55].

### TCA/acetone protein precipitation assays

For protein precipitation from conditioned medium, cells were seeded at 1 × 10^6^ cells in 100-mm dishes. After cells reached 80% confluence, the medium was replenished with fresh medium. After 24 h, the medium was harvested and mixed with 100% trichloroacetic acid (Sigma, St. Louis, MO) at a ratio of 4:1 (v/v). After 15 min incubation on ice, the mixture was centrifuged for 10 min at 13,000 rpm at 4°C. After completely removing the supernatant, 1 ml of ice cold acetone was added, after which the mixture was centrifuged for 5 min at 13,000 rpm at 4°C. TCA was removed by adding 500 mM Tris-HCl. The gel-like precipitates were lysed, resuspended in 1× SDS sample buffer, boiled for 10 min, resolved by SDS-PAGE, and analyzed by western blotting.

### Cell surface labeling

Cells were detached with diluted trypsin/EDTA, after which DMEM containing 10% FBS was added to inactivate the trypsin. The detached cells were washed with PBS and incubated with galectin-1 antibodies (10 μg/ml, R&D systems) in 3% (w/v) BSA/PBS for 1 hour. The cells were then washed three times with PBS and filtered through a cell strainer. Cell surface-associated fluorescence measurements were performed using a FACScan flow cytometer (Becton & Dickinson Biosciences). Data were analyzed using Cell Quest software.

### Cell proliferation and viability assays

For cell proliferation assays, cells were seeded at 2 × 10^4^ cells per well in 12-well culture plates. Cells were harvested by trypsinization every 24 h for 4 days, resuspended in 1 ml of medium, and counted in triplicate using a hemocytometer.

For cell viability assays, cells were seeded at 5 × 10^3^ cells per well in 96-well plates and exposed to the indicated concentrations of doxorubicin. Cell viability was measured by adding 20 μl of 10 mg/ml MTT (3-[4,5-dimethylthiazol-2-yl]-2,5-diphenyltetrazolium bromide) (Sigma, St. Louis, MO) to 100 μl of culture medium and incubating the mixture for an additional 3 h at 37°C. Next, the medium was removed, and the resultant formazan crystals were dissolved in dimethyl sulfoxide (Sigma). The optical density of each solution was assessed at 590 nm using a Multiscan EX spectrophotometer (Thermo, Vantaa, Finland).

### Cell cycle analyses by flow cytometry

Cells were harvested with trypsin, washed with PBS, and fixed in 100% ice cold methanol overnight at -20°C. Fixed cells were incubated in 50 μg/ml of propidium iodide (PI) in PBS supplemented with 1 mg/ml RNase for 30 min. Cell cycle analyses were performed using a FACScan flow cytometer (Becton & Dickinson Biosciences), and the data were analyzed using Cell Quest software. All experiments were repeated at least three times.

### Wound healing assays

Cells were seeded in 6-well culture plates and incubated until reaching approximately 90% confluency, after which they were serum starved overnight. Wounded areas were generated by scraping the plate monolayer with a pipette tip and cells were treated with cycloheximide (10 μM) to inhibit protein synthesis. After 24 h, wound closure was analyzed under a microscope. The wound closure rate was calculated using the following formula: (width of initial wounded area – final width of wounded area) / width of initial wounded area × 100 (%).

### Transwell migration and invasion assays

Cell migration assays were performed using 8 μm pore size transwell chambers (Corning, NY, USA). The lower chamber was filled with normal culture medium (DMEM with 10% FBS). Cells were suspended in DMEM with 1% FBS and seeded in the upper chamber. After 18 h, cells were stained with 0.5% crystal violet, and the number of cells on the bottom surface of the polycarbonate membrane was counted using an optical microscope. The same procedure was used for cell invasion assays, except that the upper chamber was filled with matrigel.

### Western blot analyses

Cells were washed once with PBS and resuspended in lysis buffer [20 mM Tris-HCl (pH 7.4), 0.1 mM EDTA, 150 mM NaCl, 1% NP-40, 0.1% Triton X-100, 0.1% SDS, 20 mM NaF, 1 mM Na_3_VO_4_, and 1× protease inhibitor (Roche, Basel, Switzerland)]. The proteins in the resultant extracts were boiled for 10 min in SDS sample buffer, separated on SDS-PAGE gels, and transferred to nitrocellulose membranes (Whatman, Dassel, Germany). After blocking with 5% skim milk in TBS-T for 1 h, the membranes were incubated overnight with the appropriate primary antibodies. Membranes were then washed once with TBS-T and incubated with horseradish peroxidase (HRP)-conjugated secondary antibodies for 2 h. Immunoreactive bands were visualized with the WEST-ZOL-plus Western Blot Detection System (iNtRON Biotechnology, Seoul, Korea).

### Immunoprecipitation assays

For immunoprecipitation (IP) assays, 300 μg of total protein lysate was incubated overnight with anti-galectin-1 antibody (1μg/ml) at 4°C. Protein G Sepharose 4 Fast Flow 50% slurry (w/v) (GE Healthcare, Piscataway, NJ, USA) was then added, after which the reactions were incubated for 3 h at 4°C. The resultant immunoprecipitates were washed three times with ice-cold PBS. After complete removal of the supernatants, the beads were resuspended in 1× SDS sample buffer, boiled for 10 min, resolved by SDS-PAGE, and analyzed by western blotting.

### Reverse transcriptase-polymerase chain reaction (RT-PCR) and quantitative real-time polymerase chain reaction (qPCR)

RNA was isolated using Trizol reagent (MRC, Cincinnati, OH), and subsequent RT-PCR was carried out using ReverTra Ace^®^ qPCR RT Master Mix (TOYOBO). Quantitative real-time PCR was performed with a SYBR FAST qPCR kit (KAPA) in a Thermal Cycler Dice (Takara, Otsu, Shiga, Japan) according to the manufacturer's instructions. The C(t) value was normalized using GAPDH as a control. The following primers were used: GAPDH (FWD: 5′-TCA GTG GTG GAC CTG ACC TGA CC-3′, RV: 5′-TGC TGT AGC CAA ATT CGT TGT CAT ACC-3′), galectin-1 (FWD: 5′-CAA CCC TCG CTT CAA CGC CCA CG-3′, RV: 5′-CGT ATC CAT CTG GCA GCT TGA CGG-3′), and survivin (FWD: 5′-CTT GGA GGG CTG CGC CTG CAC CC-3′, RV: 5′-CTG GCT CCC AGC CTT CCA GCT CCT TG-3′).

### Cell fractionation

For cell fractionation assays, cells were seeded at a density of 1 × 10^6^ cells in 100-mm dishes. Cells were harvested in cytoplasmic extraction buffer [10 mM HEPES (pH 7.9), 10 mM KCl, 0.1 mM EDTA, 0.1 mM EGTA, 1 mM dithiothreitol (DTT), and 0.5 mM PMSF] and incubated for 15 min on ice. The cells were then agitated for 10 min at 4°C, and NP-40 was added to a final concentration of 0.5%. The resultant lysates were clarified by centrifugation at 13,000 rpm for 5 min. The supernatants were collected as the cytosolic fractions. The nuclear pellets were washed three times with cold PBS and resuspended in nuclear extraction buffer [20 mM HEPES (pH 7.9), 400 mM NaCl, 1 mM EDTA, 1 mM EGTA, 1mM DTT, 1 mM PMSF], after which the homogenates were incubated for 15 min on ice. The nuclear extracts were agitated for 10 min at 4°C and then centrifuged at 13,000 rpm at 4°C. The resulting supernatants were collected as the nuclear fraction.

### Purification of recombinant human galectin-1

Using the galectin-1-WT construct as a backbone, a plasmid driving the expression of a GST-galectin-1 fusion protein (pGEX-4T-3-galectin-1) was produced using the following primers: FWD, 5′-CTA GGA TCC ATG GCT TGT GGT CTG GTC GC-3′; RV, 5′-CGC GTC GAC GTC AAA GGC CAC ACA TTT G-3′. *E. coli* BL21 (DE3) cells were then transformed with the resulting construct. Growing *E. coli* cells were then induced to produce protein by the addition of 1 mM isopropyl 1-thio-β-D-galactopyranoside and the cells were harvested after 4 h of growth and lysed by sonication. Clarified supernatants were loaded onto a glutathione Sepharose 4B column (GE Healthcare, Piscataway, NJ, USA), after which bound GST-tagged protein was eluted in elution buffer (50 mM Tris-HCl pH 9.5, 10 mM reduced glutathione). To remove the reduced glutathione from the eluate, the buffer was replaced with PBS during concentration. Next, 10 μl (10 units) of thrombin solution was added for each mg of tagged protein in the eluate, after which the mixture was incubated at room temperature for 24 h. Once digestion was completed, GST was removed using a glutathione Sepharose 4B column (BIO-RAD).

### Cell surface protein biotinylation assays

For cell surface protein biotinylation assays, cells were grown to 80% to 90% confluence in 100-mm dishes, washed twice with ice-cold PBS, and incubated for 15 min in ice-cold PBS. Cell surface proteins were biotinylated by incubating cells in PBS containing 0.5 mg/ml of EZ-LinkNHS-SS-Biotin (Thermo Scientific) for 30 minutes at 4°C. Biotinylated cells were washed twice with PBS and lysed. Biotinylated proteins were precipitated from the lysates using streptavidin-conjugated agarose beads (Invitrogen) and analyzed by western blotting with the indicated antibodies. To control for non-specific labeling of intracellular proteins, biotinylated cells were washed with 100 mM glutathione (Sigma-Aldrich) for 20 minutes at 4°C.

### Tumor tissue collection

The expression levels of galectin-1 and survivin were evaluated in 24 primary invasive breast cancer tissue samples from different individuals. Tissue specimens were randomly selected from the tissue archives of the Cancer Research Institute, Seoul National University. All tumors had been excised between 2004 and 2006, and were histopathologically confirmed. Informed consent was obtained from all participants before operation, and the study was approved by the Institutional Review Board of Seoul National University Hospital (H-0512-502-163).

### Statistical analyses

All experiments were performed in triplicate. Data from cell viability assays, cell proliferation assays, migration assays, invasion assays, cell cycle analyses, and real-time PCR assays are expressed as means ± standard deviations. Western blot data with patient samples were analyzed using GraphPad Prism version 4 for Windows. All results are expressed as mean ±SD. Error bars are sometimes smaller than symbols. Comparison of results from experimental groups versus control groups was done using student *t*-test and one-way ANOVA where appropriate. Standard deviations for all measured biological parameters are displayed in the appropriate figures.

## SUPPLEMENTARY MATERIALS FIGURES AND TABLES



## Abbreviations

STAT3: signal transducer and activator of transcription 3
ERK: extracellular signal-regulated kinase
TNBC: triple-negative breast cancer
FAK: focal adhesion kinase
HER2: human epidermal growth factor receptor 2
IAP: inhibitor of apoptosis protein
ER: estrogen receptor
PR: progesterone receptor
EGFR: epidermal growth factor receptor
JAK: Janus kinase
CRD: carbohydrate recognition domain
HIF-1α: hypoxia inducible factor-1α
Sp1: specificity protein-1
Egr-1: early growth response-1
shRNA: short hairpin RNA
