# Supplementary material for: Binding of galectin-1 to integrin β1 potentiates drug resistance by promoting survivin expression in breast cancer cells
Source: Oncotarget. 2017 Mar 15;8(22):35804–23. doi: 10.18632/oncotarget.16208 (PMC5482619; doi:10.18632/oncotarget.16208)
Supplement: Supplementary file 1 [file oncotarget-08-35804-s001.pdf]

Binding of galectin-1 to integrin β1 potentiates drug resistance by promoting survivin expression in breast cancer cells

Supplementary Materials

Antibodies

Antibodies specific to survivin (D-8), Actin (C-2), β-tubulin (H-235), ERK1 (K-23), p-ERK (E-4), STAT3 (H-190), integrin β1 (JB1B), HIF-1α (H-206), Egr-1 (C-19), and Sp1 (H-225) antibodies were purchased from Santa Cruz Biotechnology. Caspase-3, Src (36D10), p-Src (Tyr416), FAK, p-FAK (Tyr925), c-Raf, p-c-Raf (Ser338), MEK1/2, p-MEK1/2, c-Jun (60A8), p-STAT3 (Tyr705), p-STAT3 (Ser727), and Myc-tag (9B11) antibodies were purchased from Cell signaling Technology (Beverly, MA, USA). Galectin-1 antibody was purchased from R&D systems and FLAG-M2 monoclonal antibody was purchased from Sigma (St. Louis, MO).

Chromatin immunoprecipitation (ChIP) assays

For chromatin cross-linking, MDA-MB-231, Hs578T control shRNA, and galectin-1 shRNA cell lines (3 × 10^7cells) were harvested, washed once with PBS, and incubated in 1.42% formaldehyde for 15 min at room temperature. The cells were then lysed in a lysis buffer (10 mM Tris, pH 8.0, 10 mM NaCl, 0.2% NP-40, 1× protease inhibitor), and the resulting lysates were centrifuged for 1 min at 12,000 × *g*. Precipitated nuclei were treated with 200 U Micrococcal Nuclease (New England Biolabs, Hitchin, Hertfordshire, UK), resuspended in nuclei lysis buffer (50 mM Tris, pH 9.0, 10 mM EDTA, 1% SDS, 1× protease inhibitor), and diluted in IP dilution buffer (20 mM Tris, pH 8.0, 150 mM NaCl, 2 mM EDTA, 0.01% SDS, 1% Triton X-100, 1× protease inhibitor). The lysates were then disrupted with a sonicator (2 pulses, 30 s on/60 s off at 50% amplitude) to obtain DNA fragments of 200 to 500 bp in length (Dr. Hielscher, GmbH, Germany). Digested chromatin was immunoprecipitated with STAT3 antibody overnight at 4°C with rotation. The immunocomplexes were collected with Protein G Sepharose 4 Fast Flow 50% slurry (w/v) for 3 h at 4°C with rotation and washed sequentially as follows: once with washing buffer 1 (20 mM Tris, pH 8.0, 150 mM NaCl, 2 mM EDTA, 0.1% SDS, 1% Triton X-100), once with washing buffer 2 (10 mM Tris, pH 8.0, 0.25 M LiCl, 1 mM EDTA, 1% NP-40, 1% deoxycholate), once with 0.1× TE buffer (1 mM Tris, pH 7.6, 0.1 mM EDTA), and twice with elution buffer (0.1 M $NaHCO_3$, 1% SDS) for 5 min each. Eluted chromatin was reverse cross-linked overnight in 250 mM NaCl at 65°C, and DNA extraction was performed using phenol/chloroform and ethanol precipitation. The survivin promoter regions were PCR-amplified.

Supplementary Table 1: ER / PR / HER2 expressions in breast cancer patient samples used in the study

non-TNBC sample	ER	PR	HER2	TNBC sample	ER	PR	HER2
1	(+)	(−)	(−)	1	(−)	(−)	(−)
2	(+)	(−)	(−)	2	(−)	(−)	(−)
3	(+)	(−)	(−)	3	(−)	(−)	(−)
4	(+)	(−)	(−)	4	(−)	(−)	(−)
5	(+)	(−)	(−)	5	(−)	(−)	(−)
6	(−)	(−)	(+)	6	(−)	(−)	(−)
7	(−)	(−)	(+)	7	(−)	(−)	(−)
8	(−)	(−)	(+)	8	(−)	(−)	(−)
9	(−)	(−)	(+)	9	(−)	(−)	(−)
10	(−)	(−)	(+)	10	(−)	(−)	(−)
11	(−)	(−)	(+)	11	(−)	(−)	(−)
12	(−)	(−)	(+)				
13	(−)	(−)	(+)				

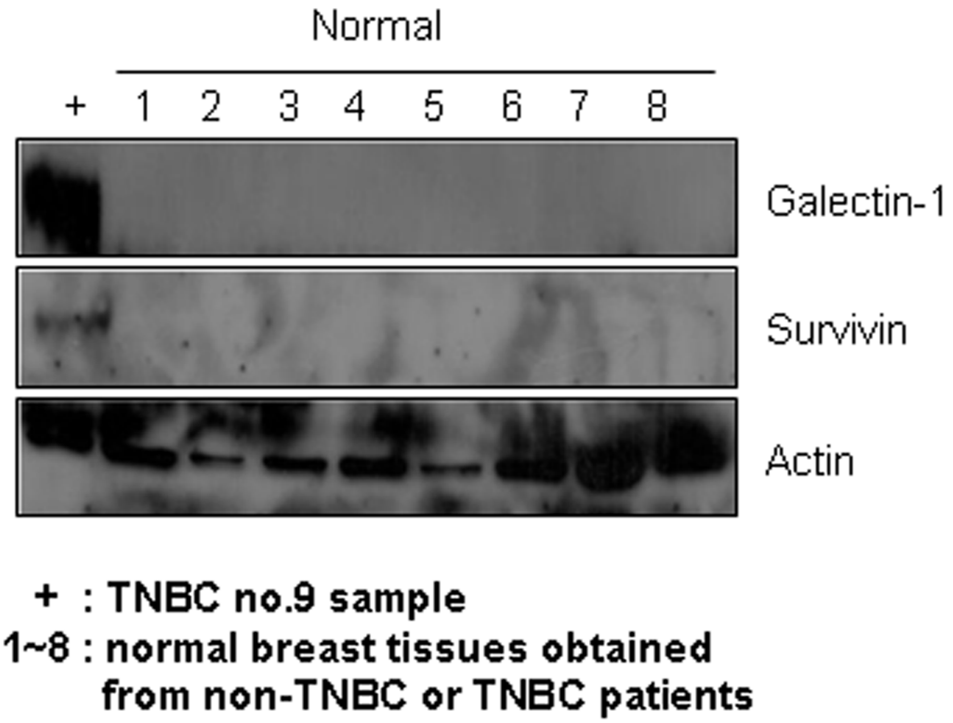

Supplementary Figure 1: Galectin-1 is not detected in normal breast tissues from non-TNBC or TNBC patients. Western blot analysis showing the expression levels of galectin-1 and survivin in lysates of normal specimens from non-TNBC or TNBC patients (n = 8). Galectin-1 and survivin expression were not detected in normal breast tissues. TNBC no.9 sample was used as a positive control.

A

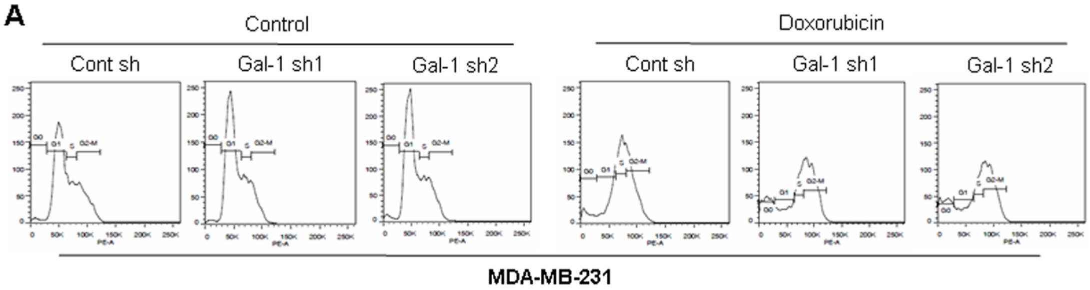

MDA-MB-231	Control			Doxorubicin		
	Cont sh	Gal-1 sh1	Gal-1 sh2	Cont sh	Gal-1 sh1	Gal-1 sh2
G2/M	17.87±4.60	18.98±6.92	11.23±1.52	52.58±3.00	53.53±3.27	43.97±3.09
S	25.94±2.18	18.98±0.39	17.29±0.60	24.07±2.55	14.71±0.78	22.24±1.50
G1	53.05±3.93	57.93±5.23	67.12±1.47	16.33±1.75	14.94±1.22	16.59±0.66
G0	3.14±0.85	4.12±1.55	4.36±0.61	7.02±1.15	16.82±1.69	17.20±2.66

B

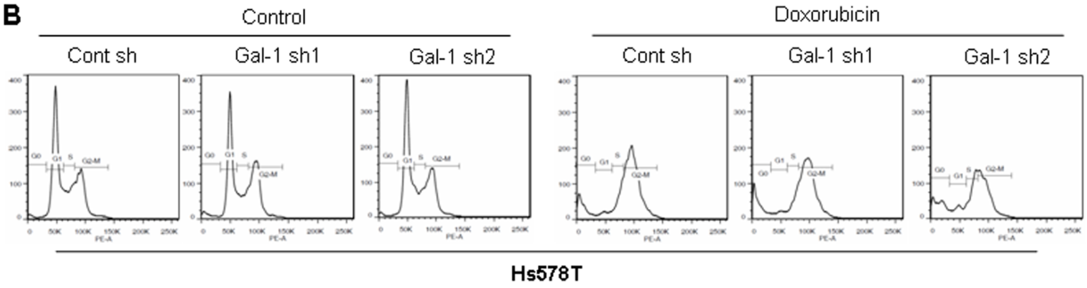

Hs578T	Control			Doxorubicin		
	Cont sh	Gal-1 sh1	Gal-1 sh2	Cont sh	Gal-1 sh1	Gal-1 sh2
G2/M	34.96±0.45	38.10±1.66	34.19±0.84	67.36±0.52	59.18±0.73	54.78±0.84
S	18.37±0.40	15.49±0.37	14.71±0.41	14.76±0.42	13.70±0.76	14.52±0.05
G1	43.17±0.53	42.68±1.41	47.66±1.68	8.17±0.84	6.58±0.60	11.07±0.81
G0	3.50±0.30	3.73±0.22	3.44±0.48	9.70±0.42	20.54±0.62	19.63±0.09

Supplementary Figure 2: Ablation of galectin-1 increases doxorubicin-induced apoptotic cell death. (**A**, **B**) Galectin-1 silenced (MDA-MB-231 Gal-1 sh1, Gal-1 sh2 and Hs578T Gal-1 sh1, Gal-1 sh2) cells and control (MDA-MB-231 Cont sh and Hs578T Cont sh) cells were seeded in 100-mm dishes at a density of 1×10^6 cells. At 24 h after cell seeding, doxorubicin was added to 0.5 µM for 48 h. Cells were fixed in methanol and incubated in 50 µg/ml propodium iodide (PI) and 1 mg/ml RNase. Histograms represent DNA from three independent experiments.

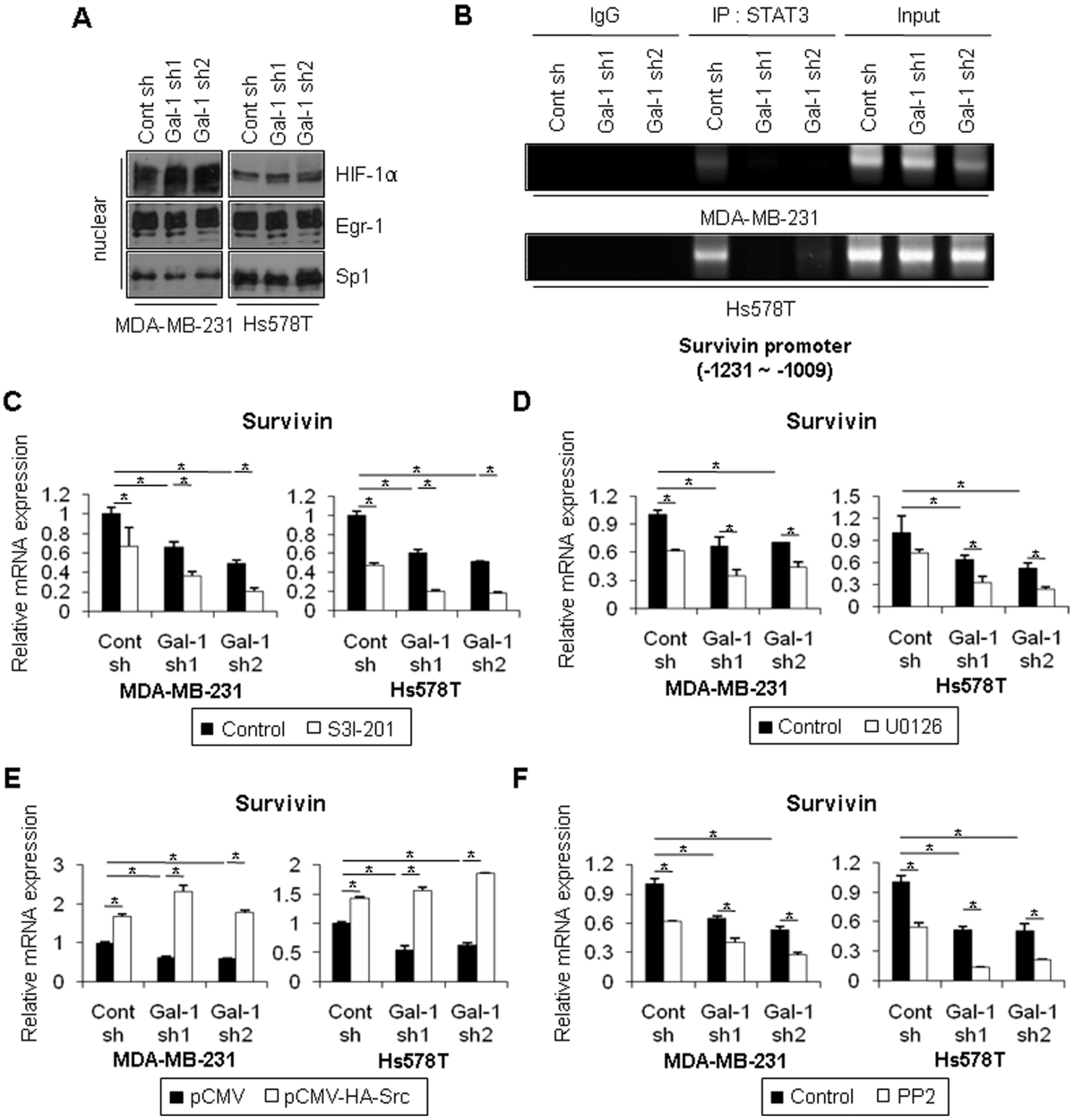

Supplementary Figure 3: Galectin-1-induced drug resistance is mediated by c-Src/survivin pathway. (**A**) To investigate the transcription factor of survivin, cell fractionation assay was performed and analyzed by western blot using antibodies specific for HIF-1α, Egr-1, Sp1. (**B**) For ChIP assay, galectin-1 silenced (MDA-MB-231 Gal-1 sh1, Gal-1 sh2 and Hs578T Gal-1 sh1, Gal-1 sh2) cells and control (MDA-MB-231 Cont sh and Hs578T Cont sh) cells were seeded at a density of 3×10^7. For chromatin cross-linking, cells were incubated in formaldehyde. The cells were then sequentially lysed with cell lysis buffer, nuclei lysis buffer, and IP dilution. The lysates were then disrupted into DNA fragments in a sonicator. Digested chromatin was immunoprecipitated with STAT3 antibody. After performing IP, beads were washed with elution buffer, and DNA extraction was carried out. The survivin promoter region was amplified by PCR. (**C**) Effect of STAT3 signaling on survivin expression. Cells were treated with STAT3 inhibitor, S3I-201 (50 μM), and subsequently analyzed by qRT-PCR analysis. qRT-PCR values were normalized with GAPDH mRNA. The results were expressed as the mean ± SD. *p* values (bar vs bar, left panel) : $p = 0.04$ (1 vs 2), $p = 0.002$ (1 vs 3), $p = 0.001$ (3 vs 4), $p = 0.0003$ (1 vs 5), $p = 0.0004$ (5 vs 6). *p* values (bar vs bar, right panel) : $p = 0.0001$ (1 vs 2), $p =$ 8.53E-05 (1 vs 3), $p =$ 6.17E-06 (3 vs 4), $p =$ 6.01E-05 (1 vs 5), $p =$ 2.3E-05 (5 vs 6). (**D**) Identification of MAPK signaling on survivin expression. Cells were treated with U0126 (10 μM) and analyzed by qRT-PCR using primer specific for survivin. qRT-PCR values were normalized with GAPDH mRNA. The results were expressed as the mean ± SD. *p* values (bar vs bar, left panel) : $p = 0.0005$ (1 vs 2), $p = 0.0002$ (3 vs 4), $p = 0.004$ (5 vs 6), $p = 0.0003$ (1 vs 3), $p = 0.0005$ (1 vs 5). *p* values (bar vs bar, right panel) : $p = 0.002$ (3 vs 4), $p = 0.0002$ (5 vs 6), $p = 0.0003$ (1 vs 3), $p = 0.0009$ (1 vs 5). (**E**) Effect of c-Src signaling on survivin expression. Galectin-1 silenced (MDA-MB-231 Gal-1 sh1, Gal-1 sh2 and Hs578T Gal-1 sh1, Gal-1 sh2) cells and control (MDA-MB-231 Cont sh and Hs578T Cont sh) cells were treated with PP2 (10 μM) or (**F**) transfected with HA-tagged c-Src construct. The cells were then analyzed by qRT-PCR using primer specific for survivin. qRT-PCR values were normalized with GAPDH mRNA. The results were expressed as the mean ± SD. *p* values (bar vs bar, E-left panel) : $p =$ 5.89E-05 (1 vs 2), $p =$ 7.62E-05 (1 vs 3), $p =$ 7.30E-05 (3 vs 4), $p =$ 2.36E-05 (1 vs 5), $p =$ 5.54E-06 (5 vs 6). *p* values (bar vs bar, E-right panel) : $p =$ 1.59E-05 (1 vs 2), $p = 0.0002$ (1 vs 3), $p =$ 5.02E-05 (3 vs 4), $p =$ 2.26E-05 (1 vs 5), $p =$ 8.24E-07 (5 vs 6). *p* values (bar vs bar, F-left panel) : $p = 0.0002$ (1 vs 2), $p = 0.002$ (3 vs 4), $p = 0.0002$ (5 vs 6), $p = 0.0004$ (1 vs 3), $p = 0.0002$ (1 vs 5). *p* values (bar vs bar, F-right panel) : $p = 0.0006$ (1 vs 2), $p =$ 2.87E-05 (3 vs 4), $p = 0.001$ (5 vs 6), $p = 0.0004$ (1 vs 3), $p = 0.0009$ (1 vs 5).

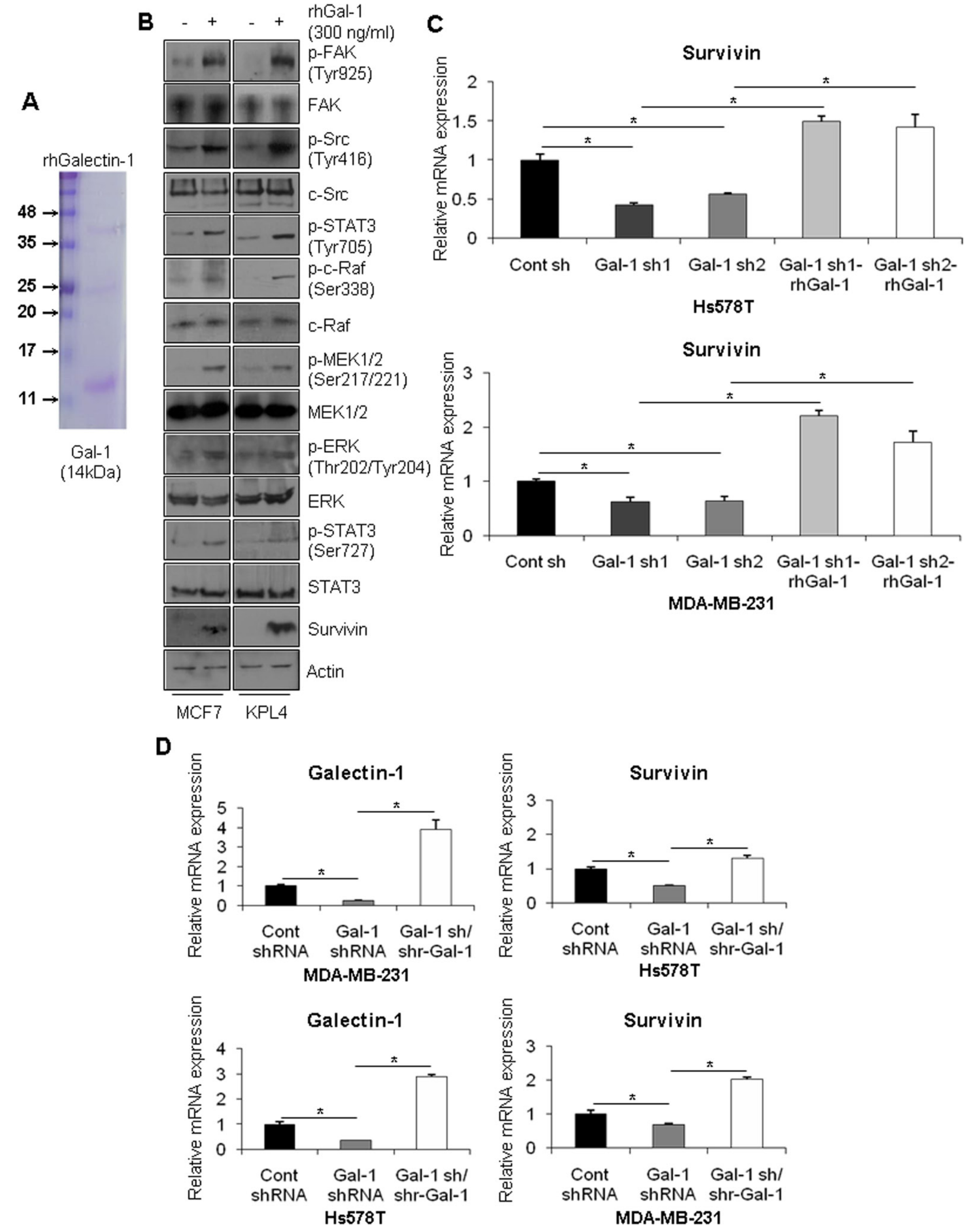

Supplementary Figure 4: Effects of extracellular galectin-1 on the expression of survivin. (**A**) Identification of galectin-1 functions as a cell surface protein. GST-tagged galectin-1 was prepared as described. To purify the recombinant human galectin-1 (rhGal-1) protein without GST, eluted protein was added 10 µl (10 units) of thrombin solution for each mg of tagged protein and incubated at room temperature for 24 h. Purified recombinant human galectin-1 (rhGal-1) protein was confirmed by Coomassie brilliant blue staining (detected to 14 kDa). (**B**) Cells were seeded in 100-mm dishes at a density of 1×10^6 cells. MCF7 and KPL4 wild-type cells were treated with recombinant human galectin-1 (rhGal-1; 300 ng/ml) for 48 h and analyzed by western blotting. (**C**) Cells were seeded in 100-mm dishes at a density of 1×10^6cells. At 24 h after cell seeding, cells were treated with recombinant human galectin-1 (rhGal-1; 300 ng/ml) protein or (**D**) pBabe-puro-myc/his-shRNA resistant galectin-1 was transfected into MDA-MB-231 Gal-1 sh1 and Hs578T Gal-1 sh1 cells for 48 h. Cells were subjected to qRT-PCR analysis. qRT-PCR values were normalized with GAPDH mRNA. The results were expressed as the mean ± SD. *p* values (bar vs bar, B-upper panel) : *p* = 0.0002 (1 vs 2), *p* = 0.0007 (1 vs 3), *p* = 1.38E-05 (2 vs 4), *p* = 0.0009 (3 vs 5). *p* values (bar vs bar, B-lower panel) : *p* = 0.002 (1 vs 2), *p* = 0.003 (1 vs 3), *p* = 3.71E-05 (2 vs 4), *p* = 0.0009 (3 vs 5). *p* values (bar vs bar, C-upper-left panel) : *p* = 5.17E-05 (1 vs 2), *p* = 0.0003 (2 vs 3). *p* values (bar vs bar, C-upper-right panel) : *p* = 0.02 (1 vs 2), *p* = 5.32E-06 (2 vs 3). *p* values (bar vs bar, C-lower-left panel) : *p* = 0.0004 (1 vs 2), *p* = 1.9E-07 (2 vs 3). *p* values (bar vs bar, C-lower-right panel) : *p* = 0.0002 (1 vs 2), *p* = 0.0002 (2 vs 3).

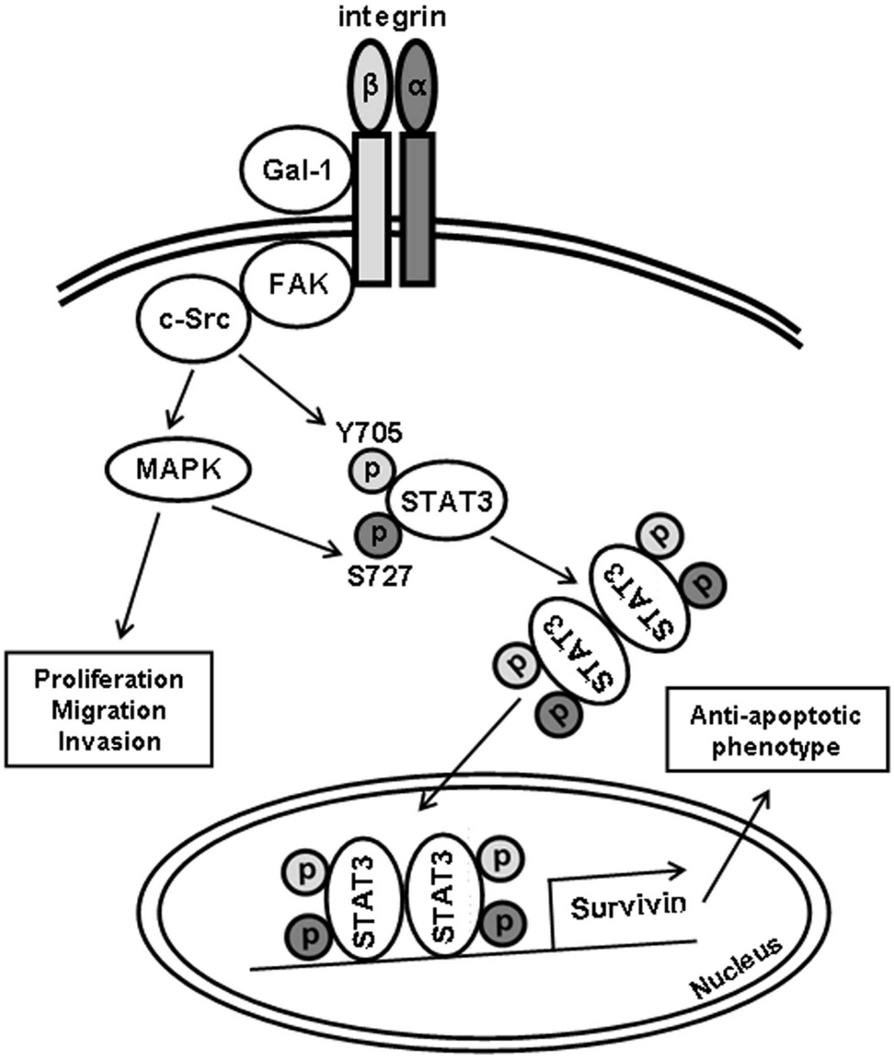

Supplementary Figure 5: Schematic representation of the galectin-1-mediated drug resistance. Ablation of galectin-1 decreases interaction with integrin β1 resulted in inhibition of the downstream FAK/c-Src signaling. The result of c-Src phosphorylation was attenuated, downstream MAPK and STAT3 phosphorylation (both Tyr705 and Ser727) was decreased in galectin-1 shRNA cells. As the phosphorylation of STAT3 was inhibited, STAT3 dimerization was blocked and followed by suppression of STAT3 translocation to the nucleus. These changes resulted in the down-regulation of survivin expression, which are important anti-apoptotic regulators, inhibiting drug resistance in human breast cancer cells.
